# Reproducible Fabrication of Perovskite Photovoltaics via Supramolecule Confinement Growth

**DOI:** 10.1007/s40820-025-01923-w

**Published:** 2025-09-15

**Authors:** Xinyi Liu, Jin Xie, Ziren Zhou, Huijun Lian, Xinyuan Sui, Qing Li, Miaoyu Lin, Da Liu, Haiyang Yuan, Feng Gao, Yongzhen Wu, Hua Gui Yang, Shuang Yang, Yu Hou

**Affiliations:** 1 Key Laboratory for Ultrafine Materials of Ministry of Education, Shanghai Engineering Research Center of Hierarchical Nanomaterials, School of Materials Science and Engineering, East China University of Science and Technology, Shanghai, 200237 People’s Republic of China; 2 Department of Physics, Chemistry and Biology (IFM), Linköping University, Linköping, Sweden; 3 Institute of Fine Chemicals, School of Chemistry and Molecular Engineering, East China University of Science and Technology, Shanghai, 200237 People’s Republic of China

**Keywords:** Solar cells, Reproducibility, Perovskites, Space-confined growth, Supramolecules

## Abstract

The solution processibility of perovskites provides a cost-effective and high-throughput route for fabricating state-of-the-art solar cells. However, the fast kinetics of precursor-to-perovskite transformation is susceptible to processing conditions, resulting in an uncontrollable variance in device performance. Here, we demonstrate a supramolecule confined approach to reproducibly fabricate perovskite films with an ultrasmooth, electronically homogeneous surface. The assembly of a calixarene capping layer on precursor surface can induce host–guest interactions with solvent molecules to tailor the desolvation kinetics, and initiate the perovskite crystallization from the sharp molecule–precursor interface. These combined effects significantly reduced the spatial variance and extended the processing window of perovskite films. As a result, the standard efficiency deviations of device-to-device and batch-to-batch devices were reduced from 0.64–0.26% to 0.67–0.23%, respectively. In addition, the perovskite films with ultrasmooth top surfaces exhibited photoluminescence quantum yield > 10% and surface recombination velocities < 100 cm s^−1^ for both interfaces that yielded p-i-n structured solar cells with power conversion efficiency over 25%.

**Supplementary Information:**

The online version contains supplementary material available at 10.1007/s40820-025-01923-w.

## Highlights

- Demonstrating a new concept of “supermolecule confined growth” of perovskite thin films by constructing a compact, ultraflat 4-tert-butylthiacalix[4]arene capping layer atop perovskite precursor film to engineer the perovskite formation dynamics.
- The supramolecule confined approach enabled the highly reproducible fabrication of perovskite films with a root mean square < 10 nm and electronic homogeneity, which significantly minimized the power conversion efficiency variations for both device-to-device and batch-to-batch solar cell devices.
- The obtained perovskite films exhibited photoluminescence quantum yield > 10% and surface recombination velocities < 100 cm s^−1^ for both interfaces.

**Supplementary Information:**

The online version contains supplementary material available at 10.1007/s40820-025-01923-w.

## Graphical abstract

**Figure:**
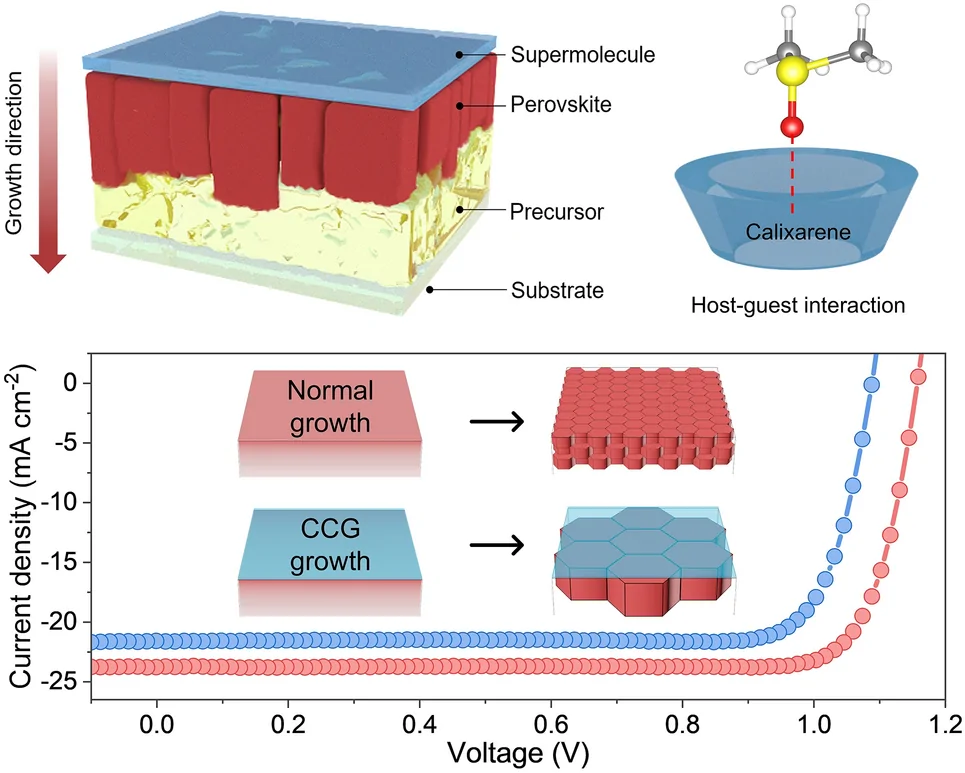

## Introduction

Lead halide hybrid perovskites possess relatively low enthalpies for phase formation [1–4], thus allowing their thin-film products to be readily fabricated under a small thermodynamic driving force. Solution deposition of high-quality perovskite films, however, requires delicate control of processing conditions, including temperature [5, 6], atmosphere [7–9], aging time [10] and passivation layer [11]. Taking antisolvent treatment as an example, the antisolvent dripping must be strictly kept in a narrow range of a few seconds [12], and the as-cast films normally need to be annealed without delay, in respect of both spin- and blade-coated ones [13]. Such rigorous formation kinetics usually renders large performance variability of individual devices from one batch (device-to-device) as well as ones from different batches (batch-to-batch), limiting the qualification rate of produced perovskite devices.

Crystallization in solution typically begins with the supersaturation phenomenon, which is highly dependent on the desolvation process. Fast annealing methods (e.g., hot casting, laser annealing and isothermal crystallization) and mechanical facilities (e.g., nitrogen knife and vacuum pump) have been used to accelerate solvent evaporation and promote precursor-to-perovskite transformation within few seconds to achieve compact films [6, 14–17]. In these representative cases, supersaturation occurs promptly after the incorporation of certain environmental perturbations, in which the experimental operation must be strictly controlled to ensure repeatability. Alternatively, slow desolvation kinetics is adopted for growing large perovskite single crystals from limited nuclei [18]. Perovskite species assembled from one interface in a slow manner can be more processing-friendly and are supposed to yield high-quality films that resemble single crystalline ones [19, 20]. Unfortunately, such slow process usually experiences strong anisotropic growth or solute depletion, which inevitably leaves rough, nonuniform surface morphology, and result in large efficiency variations. Now, only few works have reported ultrasmooth perovskite films, but together with very small grains or low crystallinity that are not suitable for the solar cell applications [21, 22]. Therefore, how to delicately control the desolvation and formation process to reproduceable fabricate highly crystalline, smooth perovskite films is a key challenge toward the production and application of perovskite solar cells (PSCs).

Herein, we engineered the perovskite formation dynamics by constructing a sharp calixarene–precursor interface that initializes the perovskite growth, instead of the air–precursor interface or bulk precursor film. The unique capping layer with molecular permeability mediates the desolvation kinetics via host–guest interactions and confines the perovskite formation beneath the flat interface, which enables the reproduceable fabrication of highly crystalline, ultrasmooth perovskite films. Inverted heterojunction solar cells based on this strategy yielded photovoltaic efficiency of 25.09% and excellent reproducibility among different devices and batches with a standard efficiency deviation of < 0.26%.

## Experimental Section

### Materials

4-*tert*-butylthiacalix[4]arene (tBTCA, > 98%), lead iodide (PbI_2_, 99.999%), phenol (99.5%), tetrabutylammonium chloride (> 98%), γ-cyclodextrin (> 99%) and polymethyl methacrylate (PMMA) were purchased from TCI. Calix[4]arene (C[4]A), 4-tert-butylcalix[4]arene (tBC[4]A, > 98%), 4-tert-butylcalix[6]arene (tBC [6]A, > 98%), 4-tert-butylcalix[8]arene (tBC[8]A, > 98%) and vancomycin hydrochloride were purchased from Macklin. Methylammonium iodide (MAI), methylammonium bromide (MABr) and formamidinium iodide (FAI) were purchased from Dyesol. Lead bromide (PbBr_2_, 99.999%), dimethyl sulfoxide (DMSO, 99.9%), butane-1,4-diammonium iodide (BDAI_2_), polyvinylpyrrolidone (PVP) and poly[bis(4-phenyl)(2,4,6-trimethylphenyl)amine] (PTAA) were purchased from Sigma-Aldrich. *N,N*-dimethylformamide (DMF, 99.8%), chlorobenzene (99.5%) and 2-methoxyethanol (99%) were purchased from Alfa Aesar. Nickel (II) acetate tetrahydrate (Ni(CH_3_COO)_2_·4H_2_O, > 98%) was purchased from Sinopharm. [6, 6]-Phenyl-C_61_-butyric acid methyl ester (PCBM, 99.5%) and bathocuproine (BCP, 99%) were purchased from Nichem. All chemicals were used as received.

### Device Fabrication

The NiO_x_ hole transport layer (HTL) was deposited on UV-cleaned FTO substrate according to our previous work [23]. The active layers were deposited by spin-coating the perovskite precursors (159 mg MAI and 461 mg PbI_2_ in 560 μL DMF and 80 μL DMSO) on HTL at 1000 r min^−1^ for 5 s and 4000 r min^−1^ for 30 s, during which 120 μL pure or tBTCA-containing chlorobenzene were dripped to remove the solvent at 23 s prior to the end. All CCG samples were fabricated using chlorobenzene solutions containing 60 mM tBTCA, except where capping layer concentrations were comparatively characterized. All as-deposited perovskite films were annealed at 100 °C for 10 min in nitrogen glove box. The FA_0.8_MA_0.2_Pb(I_0.8_Br_0.2_)_3_-based absorber was fabricated according to the literature [24]. The Cs_0.05_FA_0.81_MA_0.14_PbI_2.85_Br_0.15_-based absorber was fabricated by spin-coating the perovskite precursors (18 mg CsI, 22 mg MABr, 2.5 mg PbBr_2_, 195 mg FAI and 642 mg PbI_2_ in 800 μL DMF and 200 μL DMSO) on HTL at 1000 rpm for 5 s and 4000 rpm for 20 s, and the pure or tBTCA-containing chlorobenzene was dripped to remove the solvent at 20 s prior to the end. After cooling down, PCBM (20 mg in 1 mL chlorobenzene) and BCP (0.5 mg in 1 mL ethanol) solutions were sequentially spin-coated on perovskite film at 2000 and 4000 rpm for 45 s, respectively, and were dried at 70 °C for another 15 min. Finally, 100 nm Ag/Au was thermally evaporated as metal electrodes.

### Materials/Film Characterization

The morphology and elemental distribution of perovskite films were characterized by field emission SEM (HITACHI S4800) and transmission electron microscope (TEM). Cross section focused ion beam (FIB) sample lamellae were prepared on a Helios G4 UC instrument. TEM characterization was performed on a ThermoFisher Talos F200X microscope under 200 kV. High-angle annular dark field (HAADF)-STEM images were recorded using a convergence semiangle of 11 mrad, and inner and outer collection angles of 59 and 200 mrad, respectively. Energy-dispersive X-ray spectroscopy (EDS) was carried out using 4 in-column Super-X detectors. XRD patterns were obtained by powder X-ray diffractometer (Bruker Advance D8, Cu Kα radiation, 40 kV). Fourier transform infrared (FTIR) spectroscopy was performed using FTIR Nicolet is50 spectrometer. A Cary 500 UV–Vis–NIR spectrophotometer was applied to measure the optical absorption spectra of perovskite films. Photoluminescence (PL) and time-resolved PL (TRPL) spectra were characterized by Fluorolog-3-p spectrophotometer. Photoluminescence quantum yields (PLQYs) of the perovskite thin films were recorded by a Fluorolog system with a barium sulfate-coated integrating sphere. X-ray photoelectron spectroscopy (XPS, PHI5300, Mg anode, 250 W, 14 kV) was applied to characterize the composition and molecular interaction of perovskite film surface. The energy level of perovskite film surface was measured by UPS (Axis Ultra DLD, Al/Mg Kα, 450 W, 15 kV) with a He source of incident energy of 21.21 eV. AFM (Bruker) equipped with electrical preamplifier was used to simultaneously characterize the morphology and lateral electrical conductivity of perovskite films. KPFM images were collected on a SPA400 SPM (Seiko Instrument Inc). The concentration of Pb and S elements was analyzed by an inductively coupled plasma optical emission spectrometer (ICP-OES, NexION 2000-(A-10)). The perovskite precursor films were dissolved in HCl solution right after spinning for the test.

### Device Characterization

Solar cells were illuminated by a solar light simulator (Solar IV-150A, Zolix), and the light intensity was calibrated by a standard Newport calibrated KG5-filtered Si reference cell. The *J–V* curves of solar cells were measured by a Keithley 2400 digital sourcemeter with a scan rate of 0.1 V s^−1^ (scan range: − 0.1 to 1.3 V) in ambient (relative humidity: 10%–5%). The devices were masked with a metal aperture to define the active area as 0.0625 cm^2^. The transient photocurrent and photovoltage decay curves of solar cells were measured by digital storage oscilloscope (KEYSIGHT, DSOX3104T) under the excitation of pulsed laser (532 nm, DPS-532-A, 1 mJ). EQE spectra of solar cells were characterized by SCS600-QEDZ-JN system (Zolix). Frequency-dependent capacitance was measure by a LCR meter (Agilent, E4980A) to determine the trap density of states of solar cells.

### Theoretical Calculation

First-principles calculations were performed to investigate the interactions between tBTCA and perovskite surface. DFT calculations were carried out with the Vienna Ab-initio Simulation Package (VASP) [25, 26], with a projector augmented wave (PAW) atom potentials and the Perdew–Burke–Ernzerhof (PBE) exchange–correlation functional implemented [27, 28]. The kinetic energy cutoff for the plane-wave basis set was 450 eV, and the energy and force convergences were less than 10^−5^ eV and 0.01 eV Å^−1^. k-point of 1 × 1 × 1 was used for structure optimization and 2 × 2 × 1 for static calculation and work function. The empirical correction in Grimme’s scheme was used to describe the van der Waals interactions [29]. For the perovskite slab model, we chose the MAPbI_3_(001) surface consisting of 2 × 2 supercell with four slabs. The bottom two layers were fixed, whereas the top two layers and the adsorbents were fully relaxed during all the calculations. The absorption energies of all adsorbates are calculated as:

1 $$\Delta E_{ad} = E_{{\frac{ad}{{surf}}}} - E_{surf} - E_{ad}$$

where *E*_surf_, *E*_ad_ and *E*_(ad/surf)_ are the total energies of the clean MAPbI_3_ surface, the energies of the adsorbate and the surface with adsorbate, respectively. The work functions of these models were calculated with the energy difference between the Fermi and vacuum levels.

The work function of the system will be altered upon the adsorption of the TCA molecule onto the perovskite surface. This change in the interfacial dipole work function, denoted as ΔW, can be attributed to the surface dipole Δμ. The relationship between the change in the surface work function and the interfacial dipole can be elucidated as follows:

2 $$\Delta W = W_{molecule - perovskite} - W_{perovskite} = \frac{e\Delta \mu }{{\varepsilon_{0} A}}$$

where A is the surface area of per adsorbed molecule. Δμ represents the vertical component of the dipole, as it is directly related to the change of work function [30]. It is worth noting that the interfacial dipole is primarily composed of two components. The first component, μ_molecule_, arises from the molecular dipoles, while the second component, μ_chem_, stems from the chemical interaction between the molecule and the metal surface. The relationship between these two components can be described as follows:

3 $$\mu_{chem} = \Delta \mu - \mu_{molecule}$$

μ_molecule_ is obtained from a separate calculation on a freestanding molecule without the presence of a metal slab, and the structure is based on the previous structural optimization.

The dipoles and electrostatic potentials of the molecular tBTCA were calculated using the Gaussian 09W method [31]. The theoretical approach is based on the density functional theory approach using B3LYP [32]. Global optimization with frequency calculation was performed using the 6–31 g basis [33].

## Results and Discussion

### Thin Film Growth Under Molecule Confinement

**Fig. 1:**
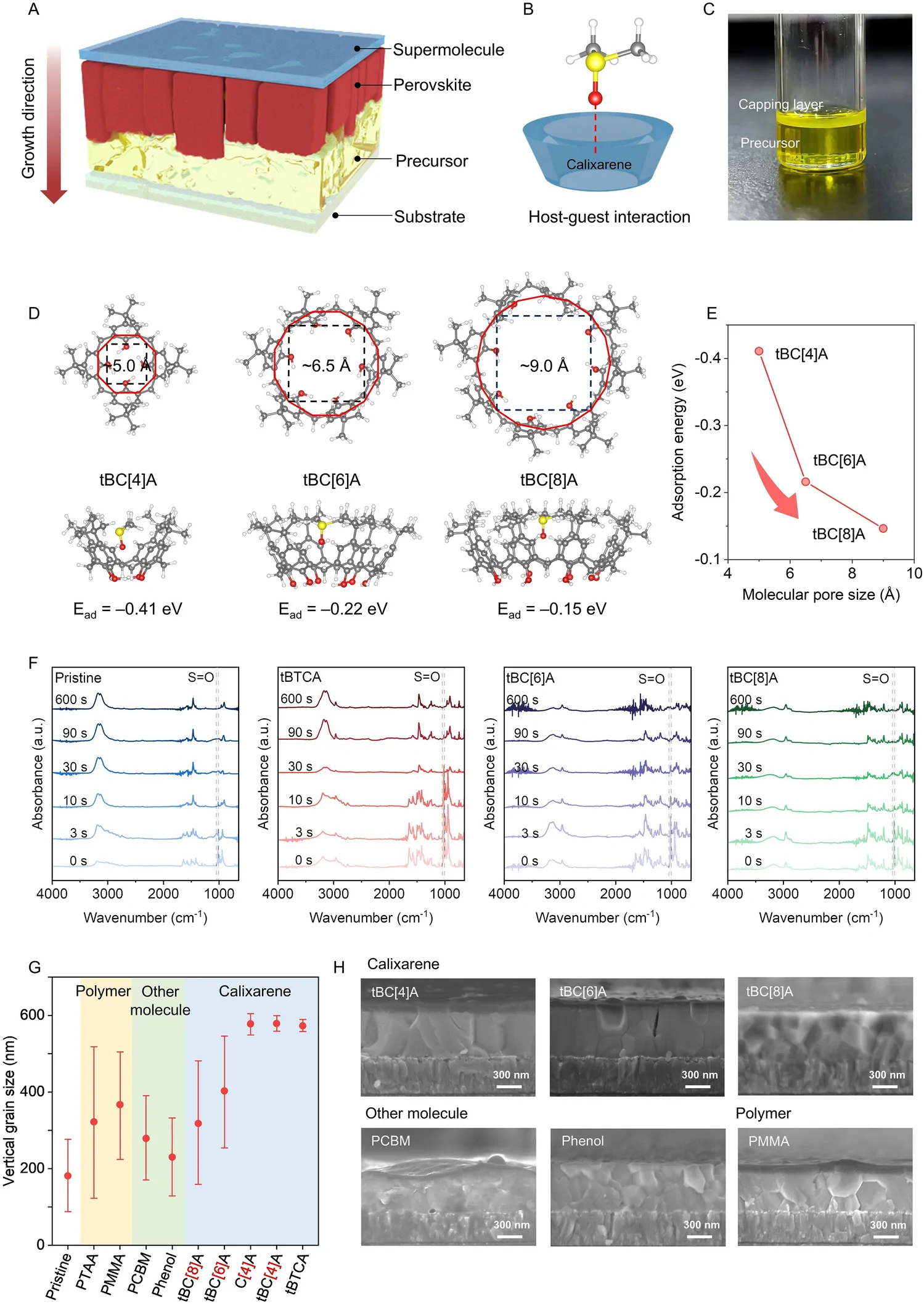
**A** Schematic illustration of perovskite film formation under capping layer. **B** Schematic host–guest interaction between calixarene and solvent molecules. **C** Photograph of perovskite precursor solution before and after dropwise addition of tBTCA chlorobenzene solution. **D** Molecular structure and macrocycle projection of calixarene molecules and the geometrically optimized adsorption configuration of tBC[4]A, tBC[6]A and tBC[8]A adsorbed with DMSO. **E** Statistical E_ad_ as a function of calixarene molecular pore sizes. **F** Evolution of FTIR spectra of different precursor films relative to annealing duration. The annealing temperature is 105 °C. **G** Statistical vertical grain sizes of perovskite films prepared by using different capping layers. **H** Cross-sectional SEM images of perovskite films deposited by using different capping layers

The schematic illustration of molecule-confined fabrication of perovskite films is shown in Fig. 1A. Macrocyclic calixarene have been shown to form host–guest complexes with small molecules, like dimethyl sulfoxide (DMSO), halobenzene and N-methylquinuclidinium ion, in previous studies [34–36]. When DMSO molecules approach the calixarene layer, it can be captured by the macrocycle via the host–guest interactions and form supramolecular structures, which retain a high DMSO content in precursor films to control the desolvation and formation process (Figs. 1B, C). The density functional theory (DFT)-derived host–guest binding energetics demonstrated an inverse relationship with the molecular pore of 4-tert-butylcalix[4]/[6]/[8]arene molecules (tBC[4]/[6]/[8]A), where enhanced DMSO binding affinity(− 0.41 eV for tBC[4]A; − 0.22 eV for tBC[6]A and − 0.15 eV for tBC[8]A) exhibited a monotonic decrease trend uponmacrocyclic expansion (Fig. 1D, E). The larger calixarenes with 6 or 8 phenol units has weaker host–guest interactions to confine the solvent molecules, which contribute to the relatively fast DMSO removal and perovskite formation. Besides, as the size of dimethylformamide (DMF) and DMSO is small, the molecular cavity or interspace of C6/8 molecular solids is not likely to block these molecules (Figs. 1D and S1). These variations further determine the following nucleation and growth of perovskites, and give rise to very different grain size of the resultant films. Scanning electron microscopy (SEM) images reveal that the vertical grain sizes are gradually reduced with the pore size of calixarene analogues (Figs. 1G, H and S2, ~ 580 nm for tBC[4]A, ~ 410 nm for tBC[6]A and ~ 300 nm for tBC[8]A), which suggests that molecules with suitable pore or interspace can sterically limit the desolvation. Meanwhile, the perovskite films prepared by smaller calixarene macrocycles generally couple with a slower crystallization kinetics (Fig. S3), which should correspond to a retarded solvent removal process.

To monitor the DMSO evaporation, we operated Fourier transform infrared spectra (FTIR) measurements of precursor films at an annealing temperature of 105 ℃. As shown in Fig. 1F, the vibrational peaks emerging at around 3150, 1470 and 1018 cm^−1^ can be assigned to the C–H stretching, CH_3_ antisymmetric bending and S=O stretching vibrations, respectively [37]. We found that the S=O peak is almost invisible at 10 s for the pristine film, indicating that the DMSO content is lower than the detection limit of the FTIR instrument. As expected, the presence of calixarene capping layer remarkably retards the DMSO evaporation: the S=O peak disappears at 90, 30 and 10 s of 4-tert-butylthiacalix[4]arene (tBTCA), tBC[6]A and tBC[8]A samples, respectively, confirming that the larger molecular inner pores allow faster solvent permeation. Similar conclusion can be obtained from X-ray diffraction (XRD) patterns, where the DMSO complexed phase lasts a longer time for precursor films prepared by using small calixarene analogues (Fig. S4).

We investigate the molecular design principle of confinement growth by systematically testing 13 more capping layers, including polymers, molecules and organic salts (Figs. 1G, H and S5, see structures in Fig. S6). First of all, it must be immiscible with DMF/DMSO to form a flat molecule–precursor interface. When tBTCA chlorobenzene solution was dropwise added to the perovskite precursor solution, a floating tBTCA layer can be obtained in spite of the miscibility between chlorobenzene and DMF (Fig. 1C). On the contrary, the PCBM solution that is partially soluble brought forth rough perovskite films because of the interdiffusion at the interface [38–40]. Another prerequisite of the capping layers is the nonreactivity with perovskites. We found that many employed materials, such as tetrabutylammonium chloride, polyvinylpyrrolidone, γ-cyclodextrin and vancomycin hydrochloride, corroded bulk perovskites (Fig. S7). These molecules or polymers generally have reactive motifs to perovskites, such as amide and carboxyl groups. In addition, the molecular permeability of capping layers does not exhibit a positive correlation with the perovskites’ grain size. An exception is the compact polymeric capping layer, like polymethyl methacrylate (PMMA) and poly[bis(4-phenyl)(2,4,6-trimethylphenyl)amine] (PTAA), whose waterproof nature would fully block the solvent percolation, and accompany with unwanted defective sites (Figs. 1G and S8). Overall, the capping layer must be compatible with perovskite precursor for stable flat interface, together with suitable molecular permeability for realizing the confinement growth.

**Fig. 2:**
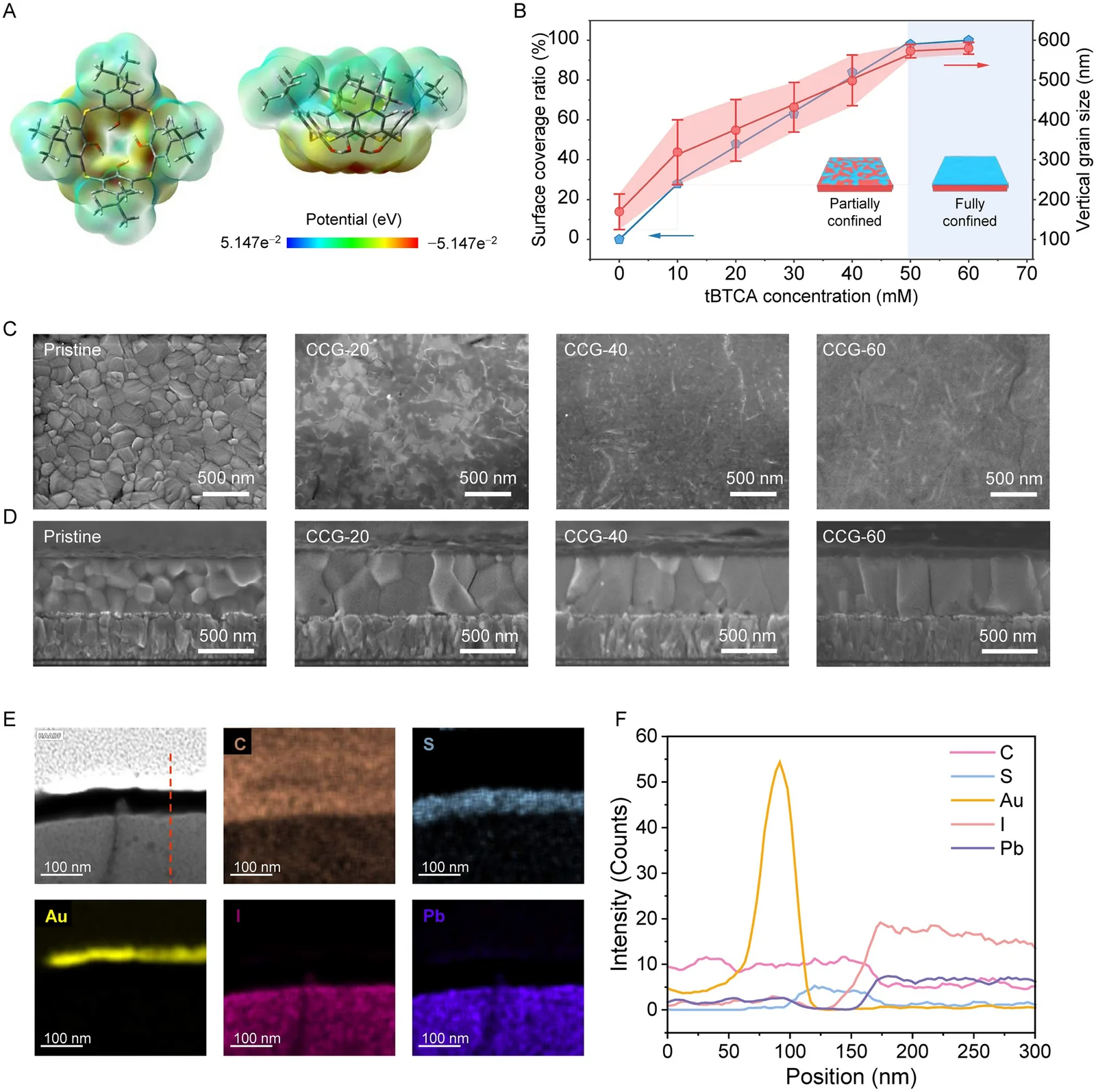
**A** Gaussian-calculated ESP maps of tBTCA, where the white, gray, red and yellow parts are H, C, O and S atoms, respectively. **B** Dependence of surface coverage ratio of the dark region and vertical perovskite grain size on the concentration of tBTCA. Insets are geometric models of perovskite films with varied molecule coverage. **C** SEM and **D** cross-sectional SEM images of tBTCA-modified perovskite films deposited on FTO/NiOx substrates. **E** Cross-sectional high-angle annular dark field (HAADF) STEM image and the associated EDS mapping of the CCG perovskite film. **F** EDS line scan taken along the indicated line in the STEM image

We selected tBTCA molecule with an appropriate pore size to regulate the perovskite crystallization process, which can not only provide an appropriate pore to accommodate DMSO with comparable diameters, but also is conducive to defect passivation. The tBTCA molecule possesses a hydroxyl and *tert*-butyl functionalized ring-shaped structure with negative charges from O- and S-donors accumulated at the lower rim of the macrocycle (Fig. 2A). The adsorption configuration of tBTCA on MAPbI_3_(001) facet is simulated by DFT method with an adsorption enthalpy (*E*_ad_) of − 1.52 eV (Fig. S9 and Table S1) [41, 42]. In this view, the tBTCA molecule should be absorbed to the Lewis acid sites of perovskite surface (e.g., undercoordinated lead atom) through its electron donating groups. This molecular binding character is thought to be beneficial for device performance by defect passivation, as has been previously observed in other macrocyclic molecules, such as crown ethers [43, 44]. Another unique feature of tBTCA is the orthogonal solubility: It is immiscible with perovskite precursor solution (Fig. S10), but can be dissolved in chlorobenzene with a solubility of > 60 mM. Hence, a capping layer can be assembled on the film surface via an antisolvent dripping procedure, whose coverage ratio and thickness can be tuned by its concentration (Fig. 2B).

SEM images reveal a typical polycrystalline morphology with a mean vertical grain size of ~ 170 nm of perovskite film treated by pure chlorobenzene (hereafter referred to as pristine, Fig. 2C, D). After introducing tBTCA, disparate dark and bright regions emerge on film surface that should be ascribed to the existence of two different phases. We noticed that the dark regions can be eliminated by chlorobenzene washing, which can be attributed to the calixarene species (Fig. S11). The coverage ratio of the dark region is about 48% upon 20 mM tBTCA, giving rise to an increased grain size of ~ 374 nm. There is an apparent transition of the films at the threshold concentration of 50 mM, beyond which all the perovskite grains become columnar structure to vertically intersect the entire film (Fig. S12). Further increment of tBTCA concentration to 60 mM leads to calixarene confined growth (hereafter referred to as CCG) of micrometer-sized columnar perovskite grains, accompanied with fully covered dark regions (Fig. 2C, D). It should be pointed out that the vertical perovskite grain size is linearly increased with the surface coverage ratio of tBTCA capping layer (Fig. S13), corroborating the changes in formation kinetics of tBTCA confined regions. To further characterize the heterointerface, CCG film structured as FTO/NiO_X_/MAPbI_3_-CCG/Au was prepared by focused ion beam (FIB) technique for scanning transmission electron microscope (STEM) characterization (Fig. 2E, F). A 70-nm-thick compact tBTCA layer can be readily distinguished on top of perovskite film with sharp interfacial boundaries. Closer inspection by energy-dispersive spectroscopy (EDS) mapping reveals distinct S- and Pb-rich regions, again confirming the stacking structure of calixarene/perovskite layers. Noteworthily, the surface of the underlying perovskite films remains flat at grain boundaries without any concave or convex microstructures, indicative of very low surface roughness.

### Film Formation Dynamic

**Fig. 3:**
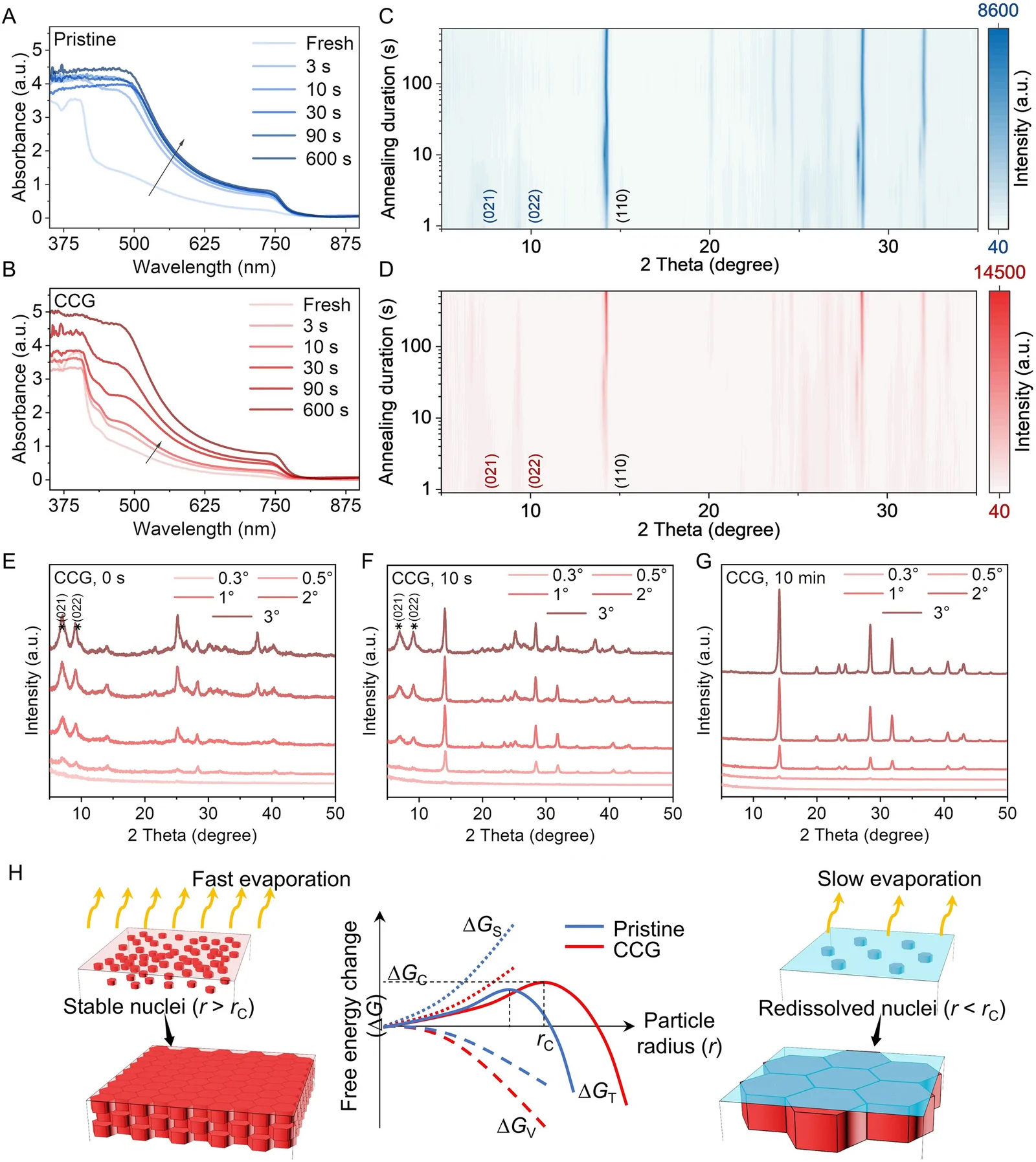
Evolution of absorption profile of **A** pristine and **B** CCG films relative to annealing duration. Evolution of XRD patterns of **C** pristine and **D** CCG films relative to annealing duration. The annealing temperature is 105 °C. **E‒G** Evolution of GIXRD patterns of incident-angle-dependent patterns of CCG film at different annealing duration with the incidence angles of 0.3°, 0.5°, 1°, 2° and 3°, respectively. **H** Schematic illustration of growth mechanism upon non- and confinement conditions

Analyzing the participation process of perovskite films is an effective way to understand the effect of tBTCA molecule on the confinement growth dynamics [45, 46]. Concerning the distinct color change of precursor films after antisolvent dripping (Fig. S14), we first evaluated the color evolution of precursor films by UV–Vis spectra. As shown in Fig. 3A, B, the optical absorbance between 400 and 750 nm of the CCG film is lower than that of the pristine one over the initial 90 s. Particularly, the absorbance at 740 nm that belongs to the characteristic absorption of perovskite phase illustrates the retarded perovskite crystallization beneath the calixarene layer (Fig. S15). Figure 3C, D shows the evolution of XRD patterns relative to the annealing duration, in which the growth rate of MAPbI_3_(110) was analyzed by Sharp–Hancock presentation. The as-determined slope *n*_1_ of 0.652 for CCG film is lower than that of 0.826 for pristine film over the initial 30 s (Fig. S16), corresponding to the slow intermediate-to-perovskite transition of CCG film under isothermal heating. The slope *n*_2_ for pristine and CCG films are 0.143 and 0.316, respectively, suggesting that the crystallization of CCG can persist in a longer time. Actually, the integrated diffraction intensity of MAPbI_3_(110) of pristine maximizes within a few seconds, while the CCG one encounters gradually improved MAPbI_3_(110) peak till 600 s accompanied by an anomalous increment of MA_2_Pb_3_I_8_(DMSO)_2_(022) peak over the initial 30 s under annealing (Fig. S17) [47]. Furthermore, we examined the ratio of S and Pb elements in the freshly coated precursor films by inductively coupled plasma optical emission spectrometer (ICP-OES). It was found that the S:Pb ratio of CCG precursor film of 0.92 is larger than that of the pristine of 0.49 and MA_2_Pb_3_I_8_(DMSO)_2_ of 0.67, illustrating the existence of excess DMSO in CCG precursor film (Table S2). Therefore, the tBTCA confinement effect would retain more residual DMSO in precursor films after spinning and delay the MA_2_Pb_3_I_8_(DMSO)_2_ phase formation as well as the subsequent evolution to MAPbI_3_ perovskite.

Angle-resolved grazing incidence XRD (GIXRD) technique was further applied to investigate the formation mechanism of perovskites. We found that the percentages of MA_2_Pb_3_I_8_(DMSO)_2_(021)/MAPbI_3_(110) and MA_2_Pb_3_I_8_(DMSO)_2_(022)/MAPbI_3_(110) of CCG film increased gradually as the detection reaches the deeper region of film (Figs. 3E‒G and S18). It suggests that the intermediate-to-perovskite phase transition primarily initiates at the calixarene–precursor interface, rather than the bottom interface or isotropic liquid bulk. An identical conclusion was drawn by the in situ PL studies, where the PL signals of bottom surface are prominently lagged behind that of the top side as a function of annealing time (Fig. S19). Moreover, the cross-sectional SEM images of CCG precursor film display almost no discernable nuclei across the entire film, which is a signature of the slow nucleation rate and downward growth. In contrast, numerous nuclei appear throughout the entire pristine film which should be caused by the fast desolvation (Fig. S20). In fact, the tBTCA layer behaves as a barrier layer to suppress the desolvation and retains a relatively low supersaturation within the precursor film, where oversaturation and nucleation will only initialize from the molecule–precursor interface (Fig. 3H and Note S1). These stable nuclei dominate the subsequent upward coarsening and ultimately yield large perovskite grains with a long crystallization duration (Fig. S21) [48]. In contrast, the fast solvent lost in pristine films in a “burst” of nucleus within the entire films, in which the perovskite formation will finish within tens of seconds.

### Smooth Top Surface Morphology of CCG Films

We have shown the critical role of calixarene capping layer in the confinement growth of perovskite thin films, and we then characterized the structural and electronic properties of the top surface/interfaces. The height profile of the as-cast films characterized by AFM reveals that the root-mean-square (RMS) roughness of tBTCA layer is only 5.28 nm (Fig. S22A), indicative of its good film-processing ability during the solution process. Corresponding conductive AFM (c-AFM) mappings further reveal the uniform and low-current signals (< 20 pA) upon the entire scan range under a high bias voltage of 10 V. Concerning the insulating nature of tBTCA, it can be inferred that the CCG layer is uniformly and densely packed on film surface (Fig. S22B). Moreover, the water contact angle of CCG film reaches up to ~ 90° as compared with ~ 52° of pristine film (Fig. S23). After loading for 10 min, the water droplet was maintained with a large contact angle of ~ 84°, manifesting the compact nature and steric blocking function of the tBTCA layer.

**Fig. 4:**
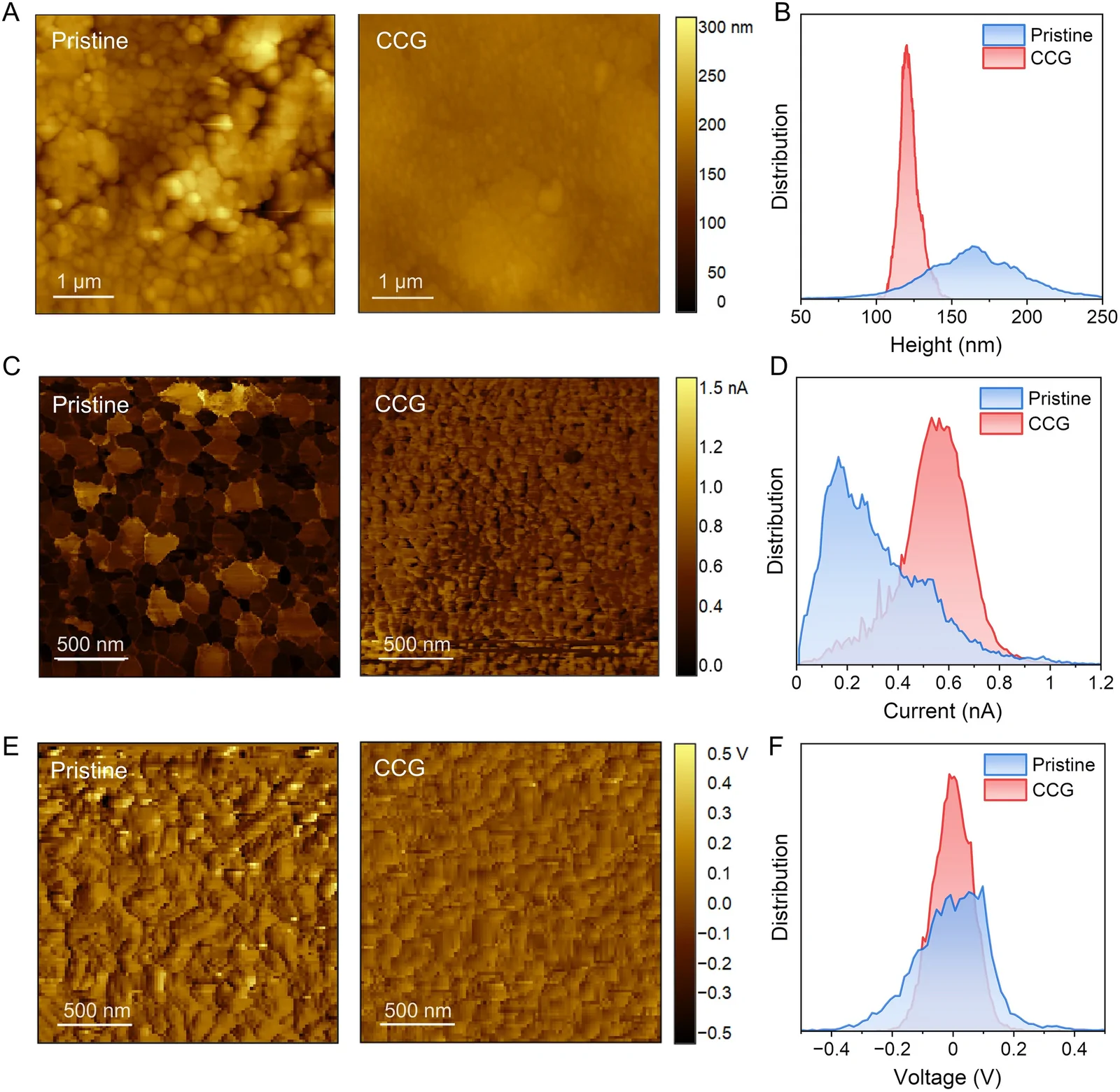
AFM images **A** and height distribution **B** of pristine and CCG films. **C** c-AFM images and **D** the corresponding current distribution of pristine and CCG films. **E** KPFM images and **F** the distribution of contact potential difference between the tip and the surface of pristine and CCG films

During the crystallization procedure, the tBTCA layer is believed to float and assemble at the liquid precursor surface, forming a flat solid–liquid interface after the removal of chlorobenzene. The aforementioned TEM, AFM and miscibility experiments have verified the formation of a sharp molecule/precursor interface with nanoscale roughness. Such unique interface should serve as a physical hindrance that spatially confines the formation of perovskite film with an ultrasmooth surface, and contributes to the narrow power conversion efficiency (PCE) distribution of solar cells. To complement our consideration, we washed the capping layer to probe the uniformity of perovskite film surface. The RMS roughness decreased from 34.15 nm of pristine to 6.79 nm of CCG film (Fig. 4A, B), which is among the best reported ones employed in PSCs. The optoelectronic homogeneity of perovskite films was then studied by c-AFM and Kelvin probe force microscopy (KPFM) techniques. As depicted in Fig. 4C, D, uniform current signals centered at ~ 0.56 nA were detected for the CCG film upon the entire scan range. Almost no notable grain boundaries can be recognized in the current mapping. By contrast, pristine film shows the varied current distribution with even > 0.5 nA change for different perovskite grains. The KPFM images of CCG perovskite films exhibited a smaller distribution, with a surface potential difference of ~ 400 mV, whereas that of pristine was > 700 mV (Fig. 4E, F). In order to investigate the impact of CCG on the reproducibility of perovskite films in different batches, we prepared several batches of perovskite films and measured the corresponding PL intensity. As seen in Fig. S24, the PL intensity of the pristine films varied and was distributed in the range of 2,000–50,000 counts, while the PL intensity of the CCG samples was basically distributed in the range of 175,000–190,000 counts, with a high degree of consistency.

On the basis of above observations, we concluded that the sharp calixarene–precursor interface should benefit the film processing and uniformity by two effects: (1) tailoring the mass transfer process as a molecular permeable layer that controls the desolvation and screens environmental stimulus; and (2) spatially confining the crystal growth beneath the interface to generate an ultraflat perovskite surface. As a consequence, the device reproductivity is expected to be improved, which will be discussed later.

### Device Performance and Characterization

**Fig. 5:**
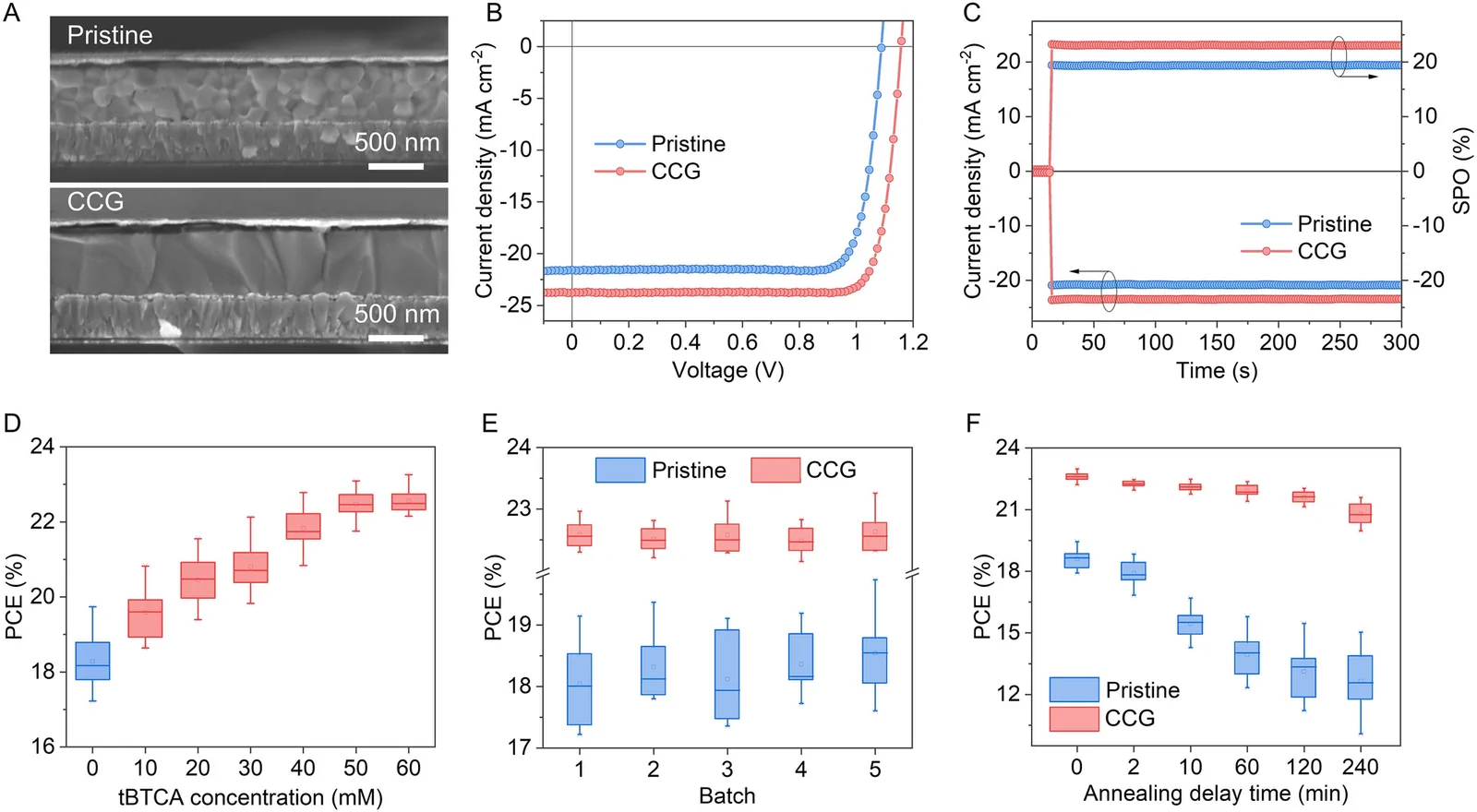
**A** Cross-sectional SEM images of pristine and CCG solar cells. **B** *J*–*V* curves of the champion solar cells based on pristine and CCG films. **C** Steady-state measurement of current density and SPO tracking of the champion pristine and CCG devices held at the fixed MPP voltages of 0.93 V and 0.98 V, respectively. **D** PCE distribution of 30 independent solar cells based on pristine and as-modified perovskite films by different concentrations of tBTCA from one batch. **E** PCE distribution of 30 independent pristine and CCG solar cells from five different batches. **F** PCE evolution of devices plotted versus annealing delay time. Each error bar represents the standard deviations of 5 individual solar cells

A p-i-n configuration (FTO/NiO_x_/perovskite/[6, 6]-phenyl-C_61_-butyric acid methyl ester (PCBM)/bathocuproine (BCP)/Ag) is used for solar cell fabrication. Cross-sectional SEM images of solar cells show that the thickness of the perovskite film is increased from ~ 490 nm for pristine device to ~ 580 nm for CCG device (Fig. 5A). The excess molecule acts as a sacrificial layer that is dissolved during the coating of PCBM layer, and leaves the bonded monolayer at perovskite/ETL interface (Fig. S25). The current–voltage (*J*–*V*) curves of solar cells were measured under simulated AM 1.5G irradiation. CCG solar cell exhibited a short-circuit current (*J*_SC_) of 23.78 mA cm^−2^, an open-circuit voltage (*V*_OC_) of 1.16 V, a fill factor (FF) of 84.53%, delivering a maximum PCE of 23.32% (Fig. 5B and Table S3). As shown in Fig. S26, there is no distinguishable photocurrent hysteresis was observed under forward and reverse sweeps. By contrast, pristine solar cell exhibited a relatively low *J*_SC_ of 21.58 mA cm^−2^, a *V*_OC_ of 1.09 V, a FF of 84.04% and a PCE of 19.77%. The stabilized power outputs (SPO) of solar cells at maximum power point (MPP) were stabilized at 19.38% for pristine device and 23.08% for CCG device (Fig. 5C). The enhanced *J*_SC_ of CCG solar cell is in accordance with the integrated *J*_SC_ from external quantum efficiency (EQE) spectra (Fig. S27). More importantly, our CCG strategy is applicable to various perovskite components, such as FA_0.8_MA_0.2_Pb(I_0.8_Br_0.2_)_3_ (denoted as FAMA) and Cs_0.05_FA_0.81_MA_0.14_PbI_2.85_Br_0.15_ (denoted as CsFAMA), and improved the champion PCE from 21.66% to 25.09% in CsFAMA cells (Figs. S28, S29 and Tables S4, S5).

Steady-state PL spectra of perovskite films show ~ 15 times enhancement of PL emission of CCG sample. Red PL emission can be clearly observed by naked eyes over the entire film surface (Fig. S30) [41]. Time-resolved PL (TRPL) spectra show the elongation of average PL lifetime (*τ*_av_) from 101.1 to 568.4 ns via the molecule confinement strategy, consistent with the steady-state PL and TPV results (Figs. S31 and S32) [49, 50]. We subsequently used TRPL spectra to explore the effect of the tBTCA layer on carrier recombination dynamics at the perovskite interface. Trioctylphosphine oxide (TOPO), as a Lewis base passivator, has been used to fully passivate the perovskite top surface with PL quantum efficiencies of over ∼90% in previous studies [51]. At this point, the remaining recombination occurs nearly all in bulk. The fitted results show bulk lifetime (τ_b_) of 1,875 ns and 2,328 ns for pristine and CCG samples, suggestive of the remarkably reduced bulk defects in perovskite. The surface recombination velocities (SRVs) of the top and bottom perovskite interface were quantified by fitting PL lifetimes as a function of the diffusion constant and film thickness (Fig. S33 and Note S2) [52, 53]. The SRVs of the CCG films in tBTCA/perovskite interface are ~ 3 times shorter than that of pristine, probably caused by the smooth surface and tBTCA adsorption. For the bottom NiO_x_/perovskite interface, the SRV reduces from 289.89 to 96.78 cm s^−1^. The reduction of trap density, especially at the shallow trap region (0.3 ~ 0.5 eV), for the CCG device was also corroborated by thermal admittance spectroscopy (Fig. S34).

It is essential to examine how the optoelectronic properties are affected by tBTCA molecules. By directly depositing a tBTCA layer on a standard perovskite film, the PL intensity was boosted by a factor of 5, indicative of its passivation function (referred as CCG-coated, Fig. S35). The PCE of solar cells also improved from 19.68% to 20.56% accordingly, albeit it is still lower than that of the CCG one of 23.32% (Fig. S36). TRPL analysis further pinpointed the reduced SRVs of top perovskite surface from 146.6 to 67.0 cm s^−1^ by depositing a tBTCA passivation layer, but the SRVs for the bottom interface of this sample are still on par with the pristine one (Fig. S37). These observations exemplify that tBTCA passivates the top surface of perovskite films, while the defect density of bulk and buried interface can be further reduced by the calixarene confined growth. In addition, the adsorption of tBTCA molecule would introduce both molecular and interfacial dipole moments opposite to the perovskite surface, which is interpreted the work function (*W*_F_) shift of − 1.02 eV as calculated by charge density difference (Figs. S38 and S39) [54, 55]. Likewise, the measured W_F_ of pristine and CCG films are 4.56 and 4.08 eV, corresponding to the valence band (VB) edge at − 5.74 and − 5.42 eV, respectively (Fig. S40). The downward band bending will energetically facilitate interfacial electron extraction, as reflected by the reduced transient photocurrent (TPC) lifetimes of CCG cells (Fig. S41) [56].

The detailed potential loss mechanism of solar cell devices was quantified by internal electron–hole quasi-Fermi level splitting (QFLS) from photoluminescence quantum yield (PLQY) results of perovskite films without and with transport layers (Note S3) [57]. The CCG perovskite film displays a high PLQY of 13.41% compared with the pristine one (1.45%), confirming the suppression of trap-assisted nonradiative recombination (Fig. S42A and Table S6). The minimized energy loss in neat CCG perovskite film delivers an implied V_OC_ of 1.233 V, about 60 mV improvement from the pristine one (Fig. S42B). The fullerene/perovskite interface has been recognized as one main channel for V_OC_ loss because of the band mismatch [58]. We found that the n-interface loss reduced from 42 to 31 mV, which can be explained by the favorable band bending by the surface-adsorbed tBTCA molecules. For the CCG device, the bulk/interface defect reduction and optimized band alignment suppress the energy loss at both bulk and interface, resulting in the improved *V*_OC_.

The light-soaking stability of unencapsulated solar cells was measured under continuous 100 mW cm^−2^ white LED illumination in a nitrogen glove box. The CCG solar cells can maintain 95.6% for their initial PCEs as a contrast to 78.8% for pristine devices after continuous illumination for 1,080 h (Fig. S43A). Subsequently, we evaluated the stability of unencapsulated devices under ambient atmosphere with controlled humidity (RH, 25 ± 3%). The CCG devices maintained over 95% of their initial efficiency after 2,000 h, demonstrating the enhanced long-term stability of devices upon CCG strategy (Fig. S43B).

### Device Performance Variation

Next, we investigated the effect of molecule confinement on the reproductivity of solar cell devices. As shown in Fig. S44, the PSCs fabricated via tBTCA confined growth delivered much better device-to-device reproducibility than the pristine cells in one batch. The standard deviations of PCEs decreased from 0.64% of the pristine cells to 0.26% of the CCG one in the same batch of devices. The narrow PCE distribution of CCG solar cells is primarily contributed by the *J*_SC_ and *V*_OC_. Our structural analysis has revealed that the grain size of perovskite film is positively correlated to the concentration of tBTCA. A similar tendency can be observed in the photovoltaic performance of solar cells, but the PCE variations remain large until the tBTCA concentration is close to 50 mM. In fact, the capping layer can fully cover the perovskite films with tBTCA concentration ≥ 50 mM and guarantees the confinement growth of the entire perovskite films at this regime (Figs. 5D, S45 and Table S3). When tBTCA concentration < 50 mM, both non- and confinement growth may occur to generate nonuniform grains and surface morphological variations. Therefore, an abrupt transition in the distribution of grain size and PCE values can be observed at tBTCA concentration of 50 mM. We subsequently tested the batch-to-batch variance of 30 individual CCG solar cells for each batch with their PCEs statistically analyzed in Fig. 5E. The PCEs of CCG devices varied between 22.15% and 23.26% with a standard PCE deviation of 0.23% by recording 5 different batches. In comparison, the efficiency of pristine ones has a large standard deviation of 0.67%, accompanied by large PCE variations between 17.22% and 19.74%.

Another advantage of the CCG method as observed is the insensitivity of device performance on the annealing delay time of precursor films by using the calixarene layer. As shown in Figs. 5F and S46, CCG solar cells are still capable to deliver the highest PCE of over 22% at the annealing delay time of 2 h at 100 °C, contrasted by the considerably decreased PCEs of pristine solar cells over the initial 10 min (Table S7). The prolonged annealing delay times should be related to the confinement effect of densely packed molecules, which initializes slow controllable crystallization from the calixarene–precursor interface, and minimizes the impact of external environments.

## Conclusions

In summary, we have developed a calixarene confinement approach that enables ultrasmooth perovskite thin films showing remarkable performance enhancement of PSCs in terms of reproductivity and efficiency. Experimental observations further elucidated that the unique calixarene layer could not only modulate the desolvation kinetics via host–guest interaction, but also physically confine the perovskite formation underneath an anticipated space. These combined effects realized the highly reproducible fabrication of ultrasmooth, uniform perovskite films with a very small RMS of 6.79 nm, which significantly minimized the PCE variations for both device-to-device and batch-to-batch solar cell devices. The as-fabricated perovskite films also present columnar-structured morphologies with > 10% PLQY and SRVs of < 100 cm s^−1^ for both top and bottom interfaces. The formation mechanism being exploited here may give an insight into the rational fabrication of high-quality perovskite thin films toward photovoltaic and other optoelectronic applications.

## Supplementary Information

Below is the link to the electronic supplementary material.

Supplementary file1 (DOCX 23460 KB)

## References

[1] D.T. Moore, H. Sai, K.W. Tan, D.-M. Smilgies, W. Zhang et al., Crystallization kinetics of organic–inorganic trihalide perovskites and the role of the lead anion in crystal growth. J. Am. Chem. Soc. **137**(6), 2350–2358 (2015). 10.1021/ja512117e 25625616 10.1021/ja512117e

[2] C.M.M. Soe, G.P. Nagabhushana, R. Shivaramaiah, H. Tsai, W. Nie et al., Structural and thermodynamic limits of layer thickness in 2D halide perovskites. Proc. Natl. Acad. Sci. U.S.A. **116**(1), 58–66 (2019). 10.1073/pnas.1811006115 30563858 10.1073/pnas.1811006115 PMC6320524

[3] D. He, P. Chen, J.A. Steele, Z. Wang, H. Xu et al., Homogeneous 2D/3D heterostructured tin halide perovskite photovoltaics. Nat. Nanotechnol. **20**(6), 779–786 (2025). 10.1038/s41565-025-01905-4 40240673 10.1038/s41565-025-01905-4 PMC12181075

[4] Z. Wu, S. Sang, J. Zheng, Q. Gao, B. Huang et al., Crystallization kinetics of hybrid perovskite solar cells. Angew. Chem. Int. Ed. **63**(17), e202319170 (2024). 10.1002/anie.202319170 10.1002/anie.202319170 38230504

[5] S. Li, Y. Xiao, R. Su, W. Xu, D. Luo et al., Coherent growth of high-Miller-index facets enhances perovskite solar cells. Nature **635**, 874–881 (2024). 10.1038/s41586-024-08159-5 39401515 10.1038/s41586-024-08159-5

[6] N. Li, X. Niu, L. Li, H. Wang, Z. Huang et al., Liquid medium annealing for fabricating durable perovskite solar cells with improved reproducibility. Science **373**(6554), 561–567 (2021). 10.1126/science.abh3884 34326239 10.1126/science.abh3884

[7] D. Liu, Y. Zheng, X.Y. Sui, X.F. Wu, C. Zou et al., Universal growth of perovskite thin monocrystals from high solute flux for sensitive self-driven X-ray detection. Nat. Commun. **15**(1), 2390 (2024). 10.1038/s41467-024-46712-y 38493199 10.1038/s41467-024-46712-y PMC10944467

[8] Y. Huang, W. Zhang, Y. Xiong, Z. Yi, C. Huang et al., Recent advancements in ambient-air fabrication of perovskite solar cells. Exploration **5**(3), 20240121 (2025). 10.1002/exp.20240121 40585769 10.1002/EXP.20240121 PMC12199439

[9] Q. Chang, P. He, H. Huang, Y. Peng, X. Han et al., Modified near-infrared annealing enabled rapid and homogeneous crystallization of perovskite films for efficient solar modules. Nano-Micro Lett. **17**(1), 272 (2025). 10.1007/s40820-025-01792-3 10.1007/s40820-025-01792-3 PMC12098230 40402386

[10] P. Wang, X. Zhang, Y. Zhou, Q. Jiang, Q. Ye et al., Solvent-controlled growth of inorganic perovskite films in dry environment for efficient and stable solar cells. Nat. Commun. **9**(1), 2225 (2018). 10.1038/s41467-018-04636-4 29884815 10.1038/s41467-018-04636-4 PMC5993712

[11] S. Wang, W. Tian, Z. Cheng, X. Shi, W. Fan et al., Fluorinated isopropanol for improved defect passivation and reproducibility in perovskite solar cells. Nat. Energy (2025). 10.1038/s41560-025-01791-z

[12] J. Xiu, B. Han, H. Gao, X. Chen, Z. Chen et al., A sustainable approach using nanocrystals functionalized green alkanes as efficient antisolvents to fabricate high-quality perovskite films. Adv. Energy Mater. **13**(28), 2300566 (2023). 10.1002/aenm.202300566

[13] Q. Li, Y. Zheng, H. Wang, X. Liu, M. Lin et al., Graphene-polymer reinforcement of perovskite lattices for durable solar cells. Science **387**(6738), 1069–1077 (2025). 10.1126/science.adu5563 40048541 10.1126/science.adu5563

[14] P. You, G. Li, G. Tang, J. Cao, F. Yan, Ultrafast laser-annealing of perovskite films for efficient perovskite solar cells. Energy Environ. Sci. **13**(4), 1187–1196 (2020). 10.1039/c9ee02324k

[15] W. Feng, X. Liu, G. Liu, G. Yang, Y. Fang et al., Blade-coating (100)-oriented α-FAPbI(3) perovskite films *via* crystal surface energy regulation for efficient and stable inverted perovskite photovoltaics. Angew. Chem. Int. Ed. **63**(39), e202403196 (2024). 10.1002/anie.202403196 10.1002/anie.202403196 38972846

[16] H. Liu, H. Wu, Z. Zhou, L. Ren, Y. Yang et al., Simultaneous mechanical and chemical synthesis of long-range-ordered perovskites. Nat. Synth. **4**(2), 196–208 (2025). 10.1038/s44160-024-00687-2

[17] Z. Yi, X. Li, Y. Xiong, G. Shen, W. Zhang et al., Self-assembled monolayers (SAMs) in inverted perovskite solar cells and their tandem photovoltaics application. Interdiscip. Mater. **3**, 203–244 (2024). 10.1002/idm2.12145

[18] L.E. Lehner, S. Demchyshyn, K. Frank, A. Minenkov, D.J. Kubicki et al., Elucidating the origins of high preferential crystal orientation in quasi-2D perovskite solar cells. Adv. Mater. **35**(5), e2208061 (2023). 10.1002/adma.202208061 36305028 10.1002/adma.202208061 PMC11475220

[19] D. Cui, X. Liu, T. Wu, X. Lin, X. Luo et al., Making room for growing oriented FASnI3 with large grains *via* cold precursor solution. Adv. Funct. Mater. **31**(25), 2100931 (2021). 10.1002/adfm.202100931

[20] J. Wang, J. Huang, M. Abdel-Shakour, T. Liu, X. Wang et al., Colloidal *zeta* potential modulation as a handle to control the crystallization kinetics of tin halide perovskites for photovoltaic applications. Angew. Chem. Int. Ed. **63**(17), e202317794 (2024). 10.1002/anie.202317794 10.1002/anie.202317794 38424035

[21] Z. Fang, B. Deng, Y. Jin, L. Yang, L. Chen et al., Surface reconstruction of wide-bandgap perovskites enables efficient perovskite/silicon tandem solar cells. Nat. Commun. **15**(1), 10554 (2024). 10.1038/s41467-024-54925-4 39632852 10.1038/s41467-024-54925-4 PMC11618607

[22] Y. Xiong, Z. Yi, W. Zhang, Y. Huang, Z. Zhang et al., Recent advances in perovskite/Cu(In,Ga)Se_2_ tandem solar cells. Mater. Today Electron. **7**, 100086 (2024). 10.1016/j.mtelec.2023.100086

[23] B. Ge, H.W. Qiao, Z.Q. Lin, Z.R. Zhou, A.P. Chen et al., Deepening the valance band edges of NiO_x_ contacts by alkaline earth metal doping for efficient perovskite photovoltaics with high open-circuit voltage. Sol. RRL **3**(8), 1900192 (2019). 10.1002/solr.201900192

[24] D. Koo, Y. Cho, U. Kim, G. Jeong, J. Lee et al., High-performance inverted perovskite solar cells with operational stability *via* n-type small molecule additive-assisted defect passivation. Adv. Energy Mater. **10**(46), 2001920 (2020). 10.1002/aenm.202001920

[25] G. Kresse, J. Hafner, *Ab initio*molecular dynamics for liquid metals. Phys. Rev. B **47**(1), 558–561 (1993). 10.1103/physrevb.47.558 10.1103/physrevb.47.558 10004490

[26] G. Kresse, J. Furthmüller, Efficient iterative schemes for *ab initio* total-energy calculations using a plane-wave basis set. Phys. Rev. B **54**(16), 11169–11186 (1996). 10.1103/physrevb.54.11169 10.1103/physrevb.54.11169 9984901

[27] G. Kresse, J. Furthmüller, Efficiency of ab-initio total energy calculations for metals and semiconductors using a plane-wave basis set. Comput. Mater. Sci. **6**(1), 15–50 (1996). 10.1016/0927-0256(96)00008-0 10.1103/physrevb.54.11169 9984901

[28] G. Kresse, D. Joubert, From ultrasoft pseudopotentials to the projector augmented-wave method. Phys. Rev. B **59**(3), 1758–1775 (1999). 10.1103/physrevb.59.1758

[29] S. Grimme, Semiempirical GGA-type density functional constructed with a long-range dispersion correction. J. Comput. Chem. **27**(15), 1787–1799 (2006). 10.1002/jcc.20495 16955487 10.1002/jcc.20495

[30] P.C. Rusu, G. Giovannetti, G. Brocks, Dipole formation at interfaces of alkanethiolate self-assembled monolayers and Ag(111). J. Phys. Chem. C **111**(39), 14448–14456 (2007). 10.1021/jp073420k

[31] M.J. Frisch et al., Gaussian 09, Revision B. 01. Gaussian, Inc., Wallingford (2010).

[32] C. Lee, W. Yang, R. Parr, Development of the *Colle*-Salvetti correlation-energy formula into a functional of the electron density. Phys. Rev. B **37**(2), 785–789 (1988). 10.1103/physrevb.37.785 10.1103/physrevb.37.785 9944570

[33] R. Ditchfield, W.J. Hehre, J.A. Pople, Self-consistent molecular-orbital methods. IX. An extended Gaussian-type basis for molecular-orbital studies of organic molecules. J. Chem. Phys. **54**(2), 724–728 (1971). 10.1063/1.1674902

[34] B. Xu, T.M. Swager, Rigid bowlic liquid crystals based on tungsten-oxo *Calix* [4] arenes: host-guest effects and head-to-tail organization. J. Am. Chem. Soc. **115**(3), 1159–1160 (1993). 10.1021/ja00056a056

[35] J. Rebek Jr., Host–guest chemistry of calixarene capsules. Chem. Commun. **8**, 637–643 (2000). 10.1039/a910339m

[36] M.O. Vysotsky, V. Böhmer, I. Thondorf, Hydrogen bonded calixarene capsules kinetically stable in DMSO. Chem. Commun. **18**, 1890–1891 (2001). 10.1039/b105613c 10.1039/b105613c 12240365

[37] N. Mozhzhukhina, L.P. Méndez De Leo, E.J. Calvo, Infrared spectroscopy studies on stability of dimethyl sulfoxide for application in a Li–air battery. J. Phys. Chem. C **117**(36), 18375–18380 (2013). 10.1021/jp407221c

[38] J. Xu, A. Buin, A.H. Ip, W. Li, O. Voznyy et al., Perovskite-fullerene hybrid materials suppress hysteresis in planar diodes. Nat. Commun. **6**, 7081 (2015). 10.1038/ncomms8081 25953105 10.1038/ncomms8081 PMC4432582

[39] C.-H. Chiang, C.-G. Wu, Bulk heterojunction perovskite–PCBM solar cells with high fill factor. Nat. Photonics **10**(3), 196–200 (2016). 10.1038/nphoton.2016.3

[40] Y. Wu, X. Yang, W. Chen, Y. Yue, M. Cai et al., Perovskite solar cells with 18.21% efficiency and area over 1 cm_2_ fabricated by heterojunction engineering. Nat. Energy **1**(11), 16148 (2016). 10.1038/nenergy.2016.148

[41] A. Amat, E. Mosconi, E. Ronca, C. Quarti, P. Umari et al., Cation-induced band-gap tuning in organohalide perovskites: interplay of spin–orbit coupling and octahedra tilting. Nano Lett. **14**(6), 3608–3616 (2014). 10.1021/nl5012992 24797342 10.1021/nl5012992

[42] S. Liu, J. Li, W. Xiao, R. Chen, Z. Sun et al., Buried interface molecular hybrid for inverted perovskite solar cells. Nature **632**(8025), 536–542 (2024). 10.1038/s41586-024-07723-3 38925147 10.1038/s41586-024-07723-3

[43] P. Ferdowsi, U. Steiner, J.V. Milić, Host-guest complexation in hybrid perovskite optoelectronics. J. Phys. Mater. **4**(4), 042011 (2021). 10.1088/2515-7639/ac299f

[44] H. Zhang, F.T. Eickemeyer, Z. Zhou, M. Mladenović, F. Jahanbakhshi et al., Multimodal host-guest complexation for efficient and stable perovskite photovoltaics. Nat. Commun. **12**(1), 3383 (2021). 10.1038/s41467-021-23566-2 34099667 10.1038/s41467-021-23566-2 PMC8185086

[45] Y. Zhu, X. Liu, X. Sui, G. Chen, Q. Li et al., Intermediate-phase homogenization through intermolecular interactions toward reproducible fabrication of perovskite solar cells. Adv. Energy Mater. **15**(29), 2500536 (2025). 10.1002/aenm.202500536

[46] J. Zhang, R. Dai, J. Yang, Y. Liu, J. Yu et al., Regulation of crystallization by introducing a multistage growth template affords efficient and stable inverted perovskite solar cells. Energy Environ. Sci. **18**(7), 3235–3247 (2025). 10.1039/D4EE06199C

[47] S. Chen, X. Xiao, B. Chen, L.L. Kelly, J. Zhao et al., Crystallization in one-step solution deposition of perovskite films: upward or downward? Sci. Adv. **7**(4), eabb2412 (2021). 10.1126/sciadv.abb2412 33523938 10.1126/sciadv.abb2412 PMC10670903

[48] X. Zheng, Y. Hou, C. Bao, J. Yin, F. Yuan et al., Managing grains and interfaces *via* ligand anchoring enables 22.3%-efficiency inverted perovskite solar cells. Nat. Energy **5**(2), 131–140 (2020). 10.1038/s41560-019-0538-4

[49] J. Wang, L. Bi, X. Huang, Q. Feng, M. Liu et al., Bilayer interface engineering through 2D/3D perovskite and surface dipole for inverted perovskite solar modules. eScience **4**(6), 100308 (2024). 10.1016/j.esci.2024.100308

[50] Y. Wang, B. Li, H. Wang, Z. Zhang, Z. Dang et al., A soft nonpolar-soluble two-dimensional perovskite for general construction of mixed-dimensional heterojunctions. Adv. Mater. **37**(14), e2419750 (2025). 10.1002/adma.202419750 40025931 10.1002/adma.202419750

[51] J. Wang, W. Fu, S. Jariwala, I. Sinha, A.K.Y. Jen et al., Reducing surface recombination velocities at the electrical contacts will improve perovskite photovoltaics. ACS Energy Lett. **4**(1), 222–227 (2019). 10.1021/acsenergylett.8b02058

[52] B. Chen, H. Chen, Y. Hou, J. Xu, S. Teale et al., Passivation of the buried interface *via* preferential crystallization of 2D perovskite on metal oxide transport layers. Adv. Mater. **33**(41), e2103394 (2021). 10.1002/adma.202103394 34425038 10.1002/adma.202103394

[53] Y. Yang, M. Yang, D.T. Moore, Y. Yan, E.M. Miller et al., Top and bottom surfaces limit carrier lifetime in lead iodide perovskite films. Nat. Energy **2**(2), 16207 (2017). 10.1038/nenergy.2016.207

[54] J. Wu, R. Zhu, G. Li, Z. Zhang, J. Pascual et al., Inhibiting interfacial nonradiative recombination in inverted perovskite solar cells with a multifunctional molecule. Adv. Mater. **36**(35), e2407433 (2024). 10.1002/adma.202407433 38973089 10.1002/adma.202407433

[55] X. Zhang, F. Liu, Y. Guan, Y. Zou, C. Wu et al., Reducing the V_oc_ loss of hole transport layer-free carbon-based perovskite solar cells *via* dual interfacial passivation. Nano-Micro Lett. **17**(1), 258 (2025). 10.1007/s40820-025-01775-4 10.1007/s40820-025-01775-4 PMC12089553 40387983

[56] H. Kanda, N. Shibayama, A.J. Huckaba, Y. Lee, S. Paek et al., Band-bending induced passivation: high performance and stable perovskite solar cells using a perhydropoly(silazane) precursor. Energy Environ. Sci. **13**(4), 1222–1230 (2020). 10.1039/C9EE02028D

[57] M. Stolterfoht, P. Caprioglio, C.M. Wolff, J.A. Márquez, J. Nordmann et al., The impact of energy alignment and interfacial recombination on the internal and external open-circuit voltage of perovskite solar cells. Energy Environ. Sci. **12**(9), 2778–2788 (2019). 10.1039/C9EE02020A

[58] F. Ye, S. Zhang, J. Warby, J. Wu, E. Gutierrez-Partida et al., Overcoming C(60)-induced interfacial recombination in inverted perovskite solar cells by electron-transporting carborane. Nat. Commun. **13**(1), 7454 (2022). 10.1038/s41467-022-34203-x 36460635 10.1038/s41467-022-34203-x PMC9718752

