# Supplementary material for: Reproducible Fabrication of Perovskite Photovoltaics via Supramolecule Confinement Growth
Source: Nanomicro Lett. 2025 Sep 15;18:67. doi: 10.1007/s40820-025-01923-w (PMC12436674; doi:10.1007/s40820-025-01923-w)
Supplement: Supplementary file 1 — Supplementary file1 (DOCX 23460 KB) [file 40820_2025_1923_MOESM1_ESM.docx]

Supporting Information for

**Reproducible Fabrication of Perovskite Photovoltaics via Supramolecule Confinement Growth**

Xinyi Liu^1, #^, Jin Xie^1, #^, Ziren Zhou^1^, Huijun Lian^1^, Xinyuan Sui^1^, Qing Li^1^, Miaoyu Lin^1^, Da Liu^1^, Haiyang Yuan^1^, Feng Gao^2^, Yongzhen Wu^3^, Hua Gui Yang^1^, Shuang Yang^1,^ * and Yu Hou^1,^ *

^1^ Key Laboratory for Ultrafine Materials of Ministry of Education, Shanghai Engineering Research Center of Hierarchical Nanomaterials, School of Materials Science and Engineering, East China University of Science and Technology, Shanghai 200237, P. R. China

^2^ Department of Physics, Chemistry and Biology (IFM), Linköping University, Linköping, Sweden

^3^ Institute of Fine Chemicals, School of Chemistry and Molecular Engineering, East China University of Science and Technology, Shanghai 200237, P. R. China

^#^ Xinyi Liu and Jin Xie contributed equally to this work.

*Corresponding authors. E-mail: [syang@ecust.edu.cn](mailto:syang@ecust.edu.cn) (Shuang Yang); [yhou@ecust.edu.cn](mailto:yhou@ecust.edu.cn) (Yu Hou)

**Supplementary Figures and Tables**


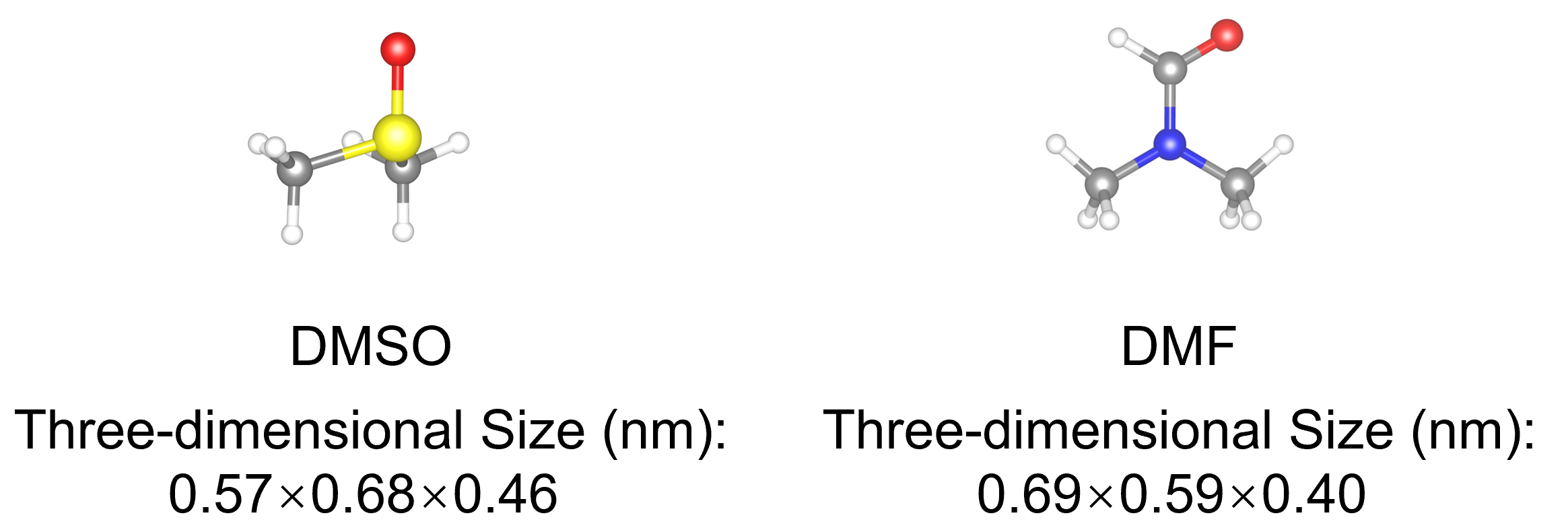


**Fig. S1** The structure and three-dimensional size of DMSO and DMF solvents


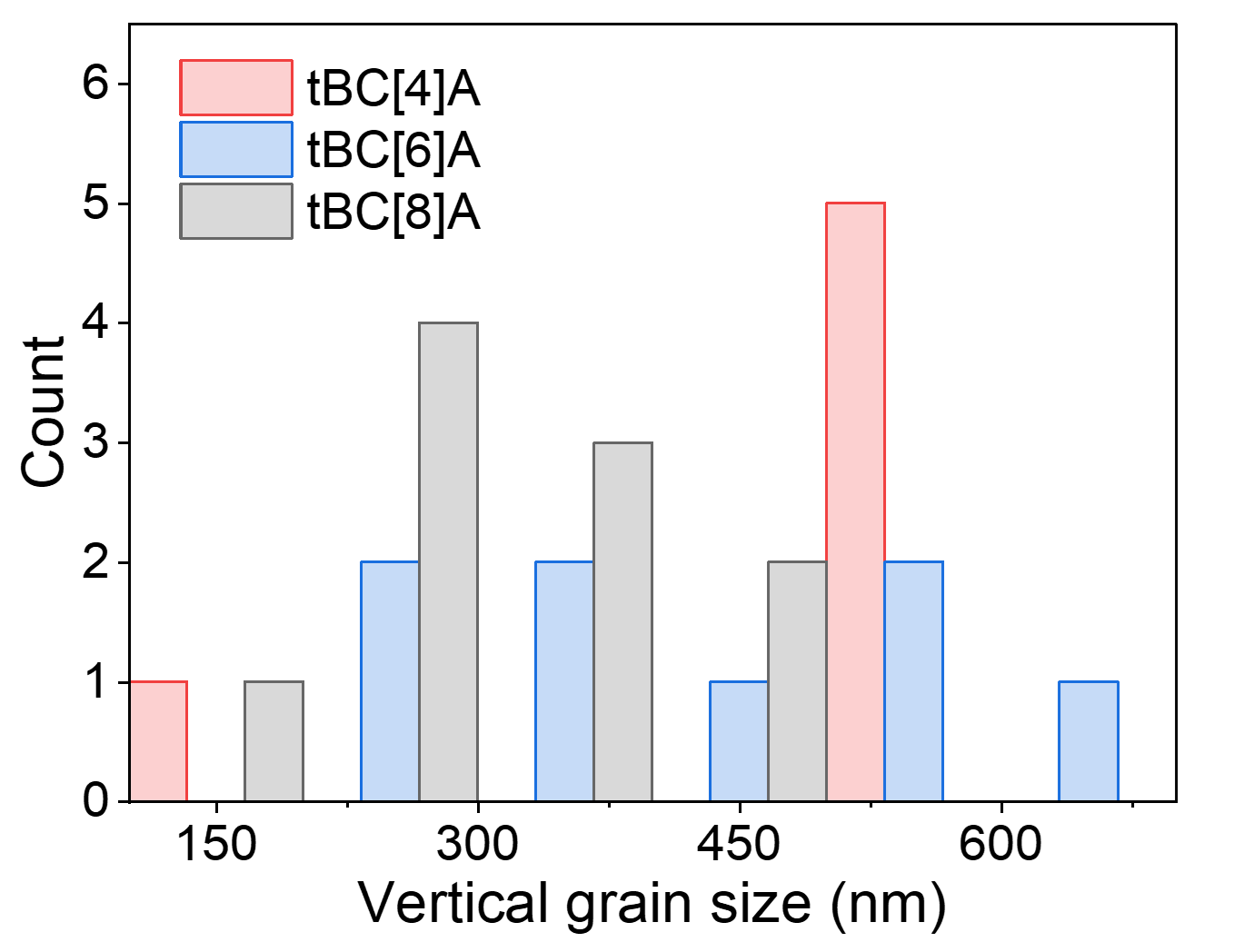


**Fig. S2** Statistical vertical grain sizes of perovskite films prepared by using tBC[4]A, tBC[6]A and tBC[8]A capping layers


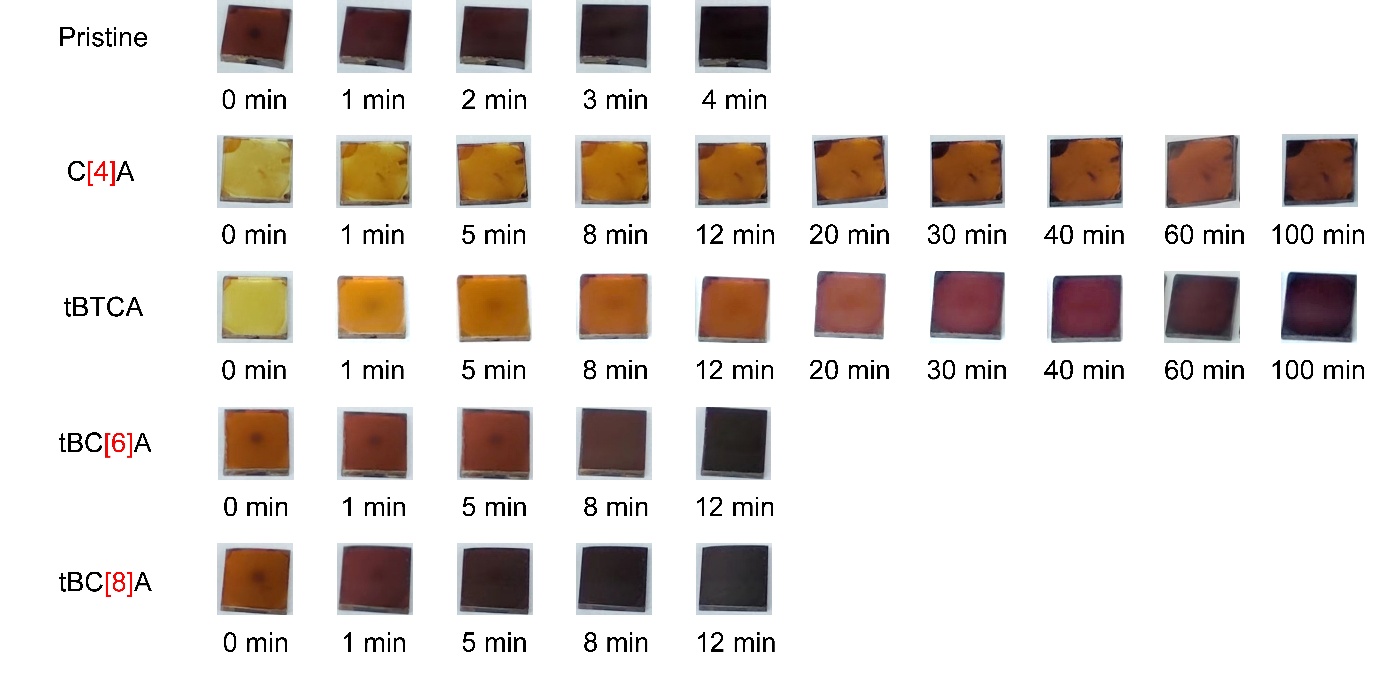


**Fig. S3** Color evolution of perovskite precursor films. All films are treated at 30 °C in N_2_ atmosphere


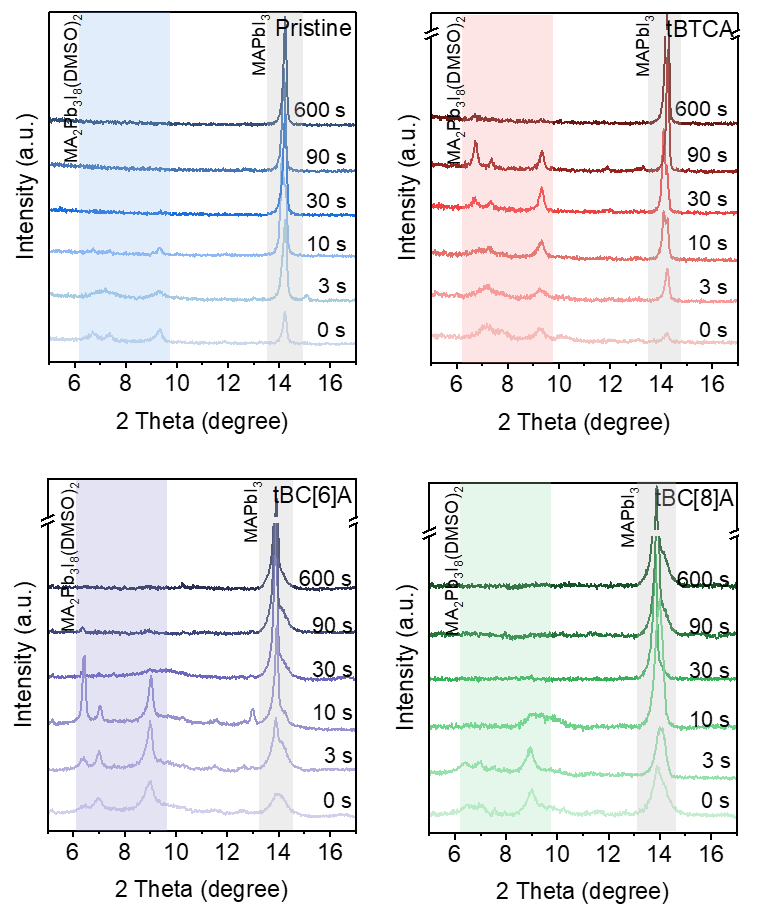


**Fig. S4** XRD patterns of different precursor films relative to annealing duration. The annealing temperature is 105 °C


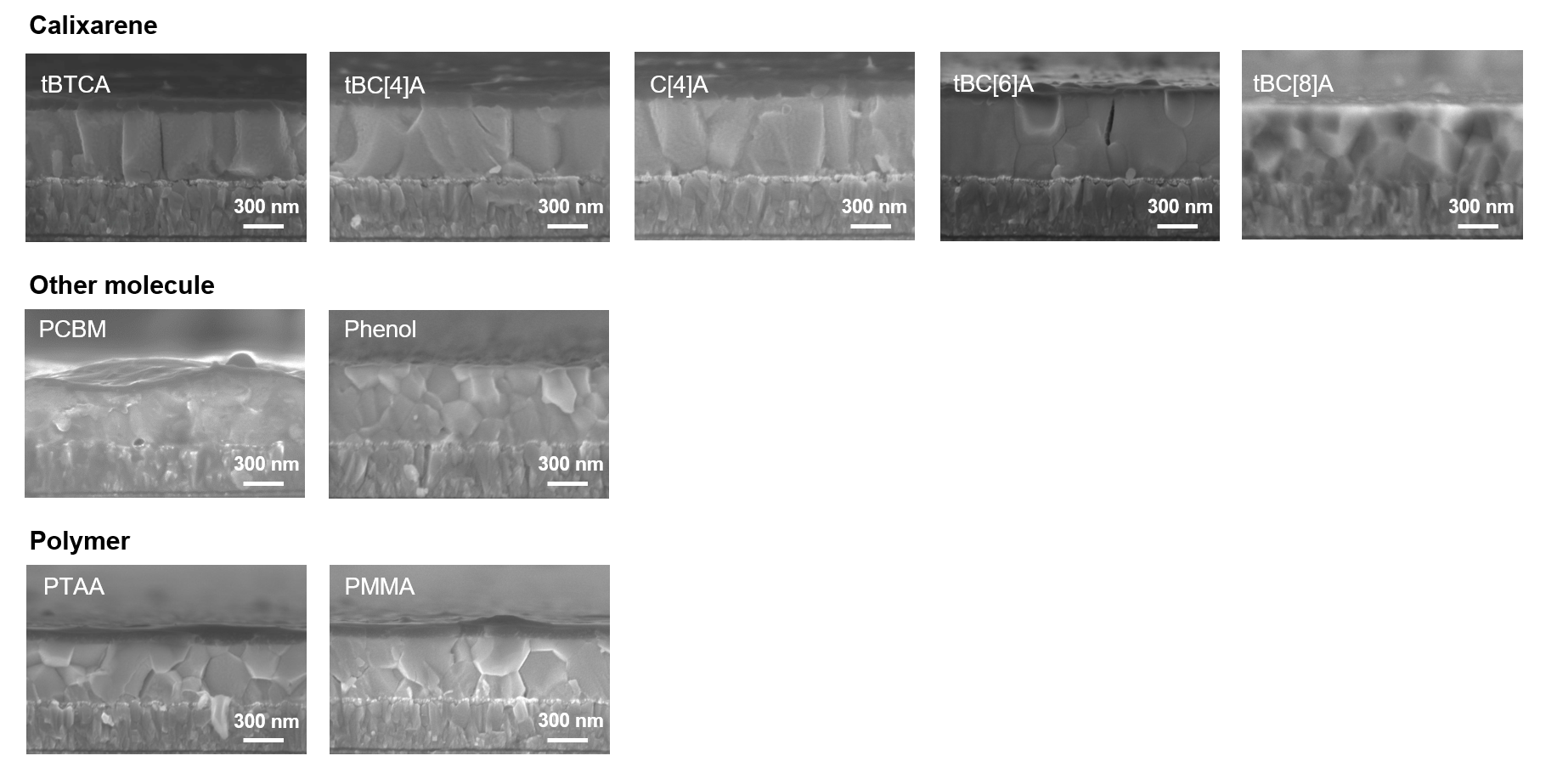


**Fig. S5** Cross-sectional SEM images of perovskite films deposited by using different capping layers


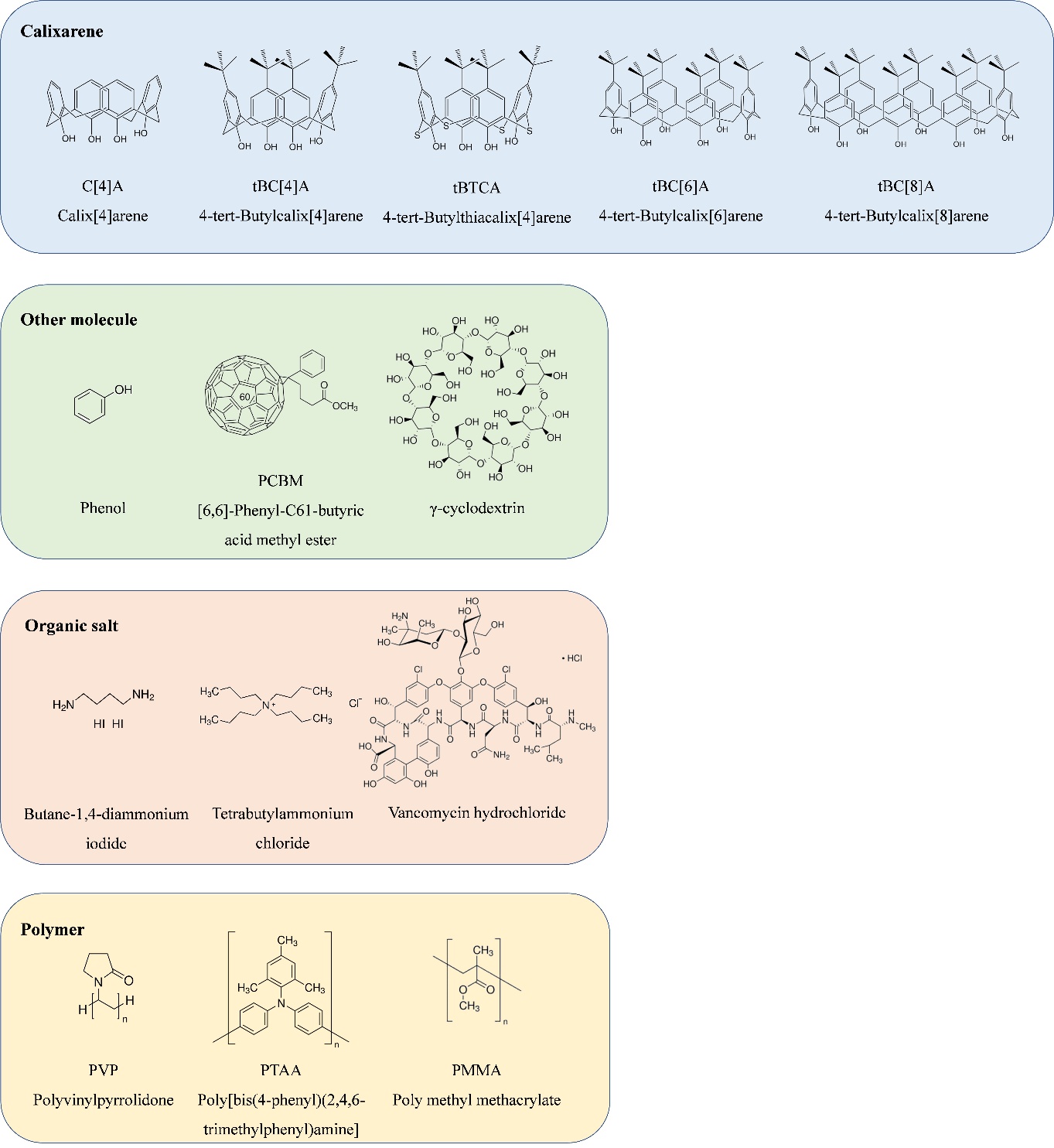


**Fig. S6** Molecule structures of the chemicals for confinement growth of perovskite thin films


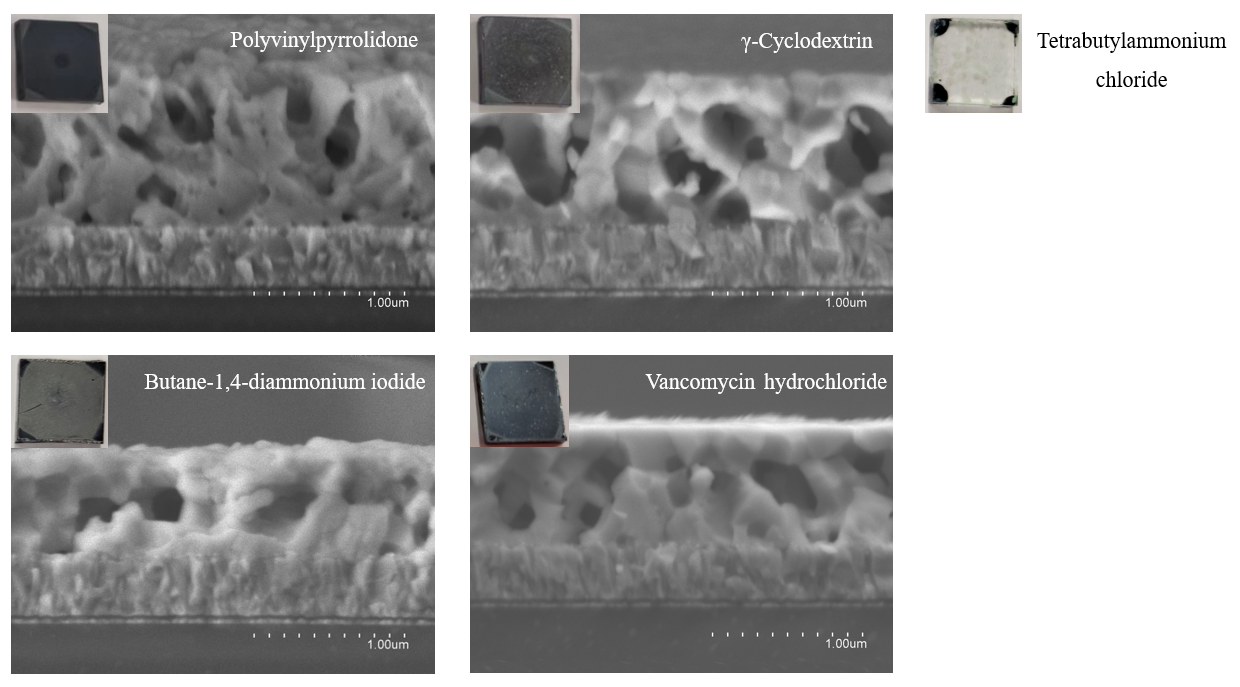


**Fig. S7** Photographs and cross-sectional SEM images of perovskite films deposited by using polyvinylpyrrolidone (PVP), γ-cyclodextrin, tetrabutylammonium chloride, butane-1,4-diammonium iodide (BDAI_2_) and vancomycin hydrochloride as capping layers. These capping layers are reactive with perovskite, and result in porous perovskite or non-perovskite films


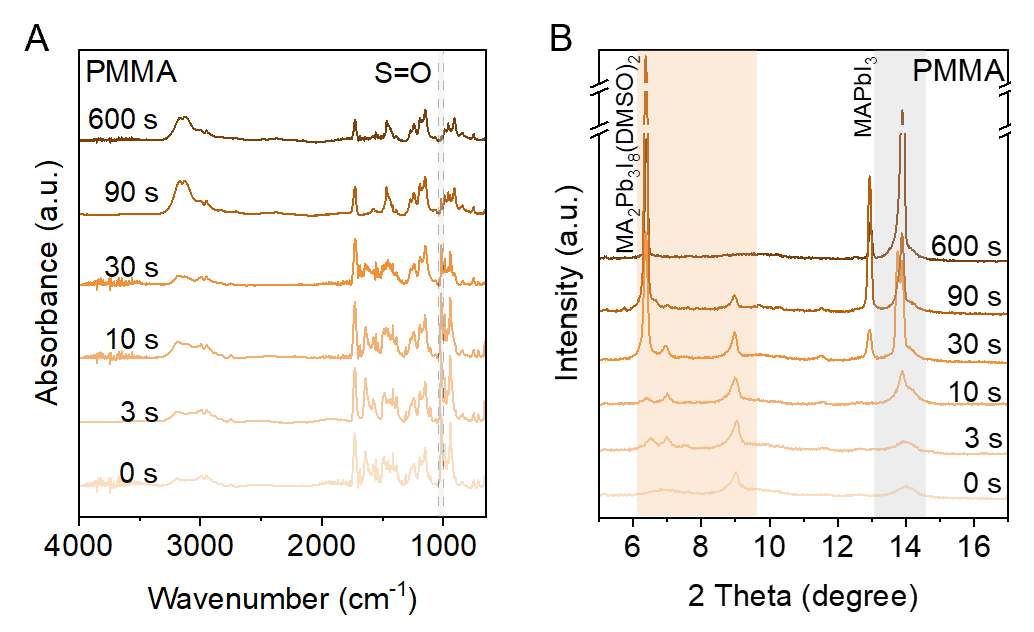


**Fig. S8** Evolution of (**A**) FTIR spectra and (**B**) XRD patterns of PMMA-treated films relative to annealing duration. The annealing temperature is 105 °C


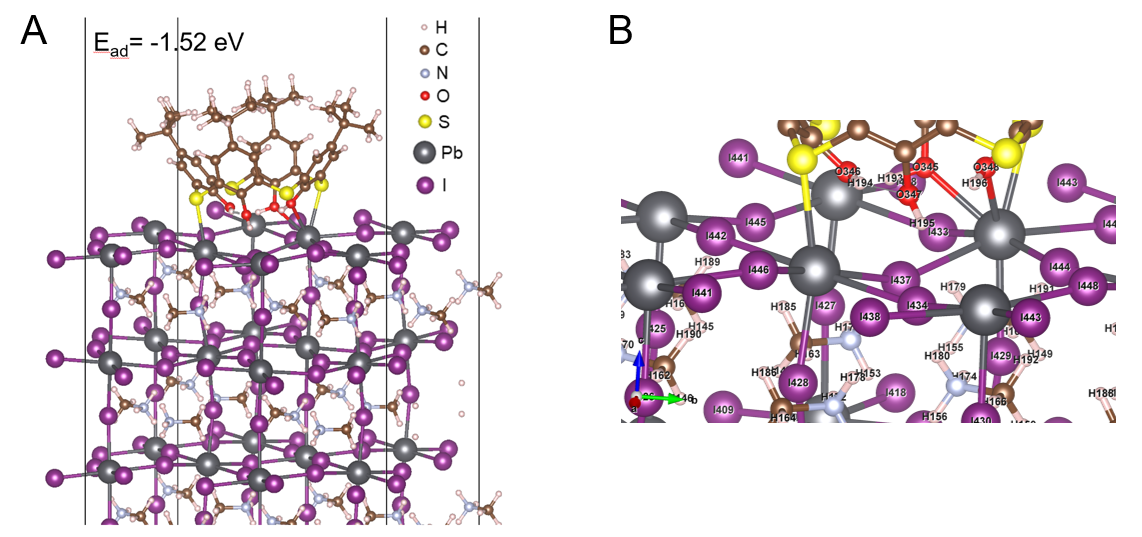


**Fig. S9** Geometrically optimized adsorption configuration of tBTCA adsorbed on MAPbI_3_(001) facet. The molecule is bonded to lead atoms through both monodentate and tridentate coordination, creating an equal number of Pb−O and Pb−S bonds. Three effective hydrogen bonds between hydroxyl group (−OH) and iodine atom can be observed (see details in Table S1)


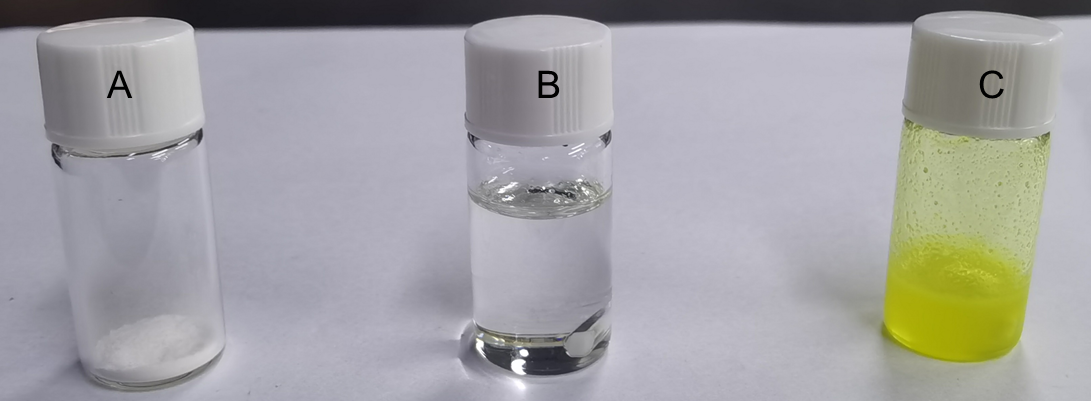


**Fig. S10** Photographs of (**A**) tBTCA powder, (**B**) tBTCA chlorobenzene solution (60 mM) and (**C**) perovskite precursor mixed with tBTCA powder


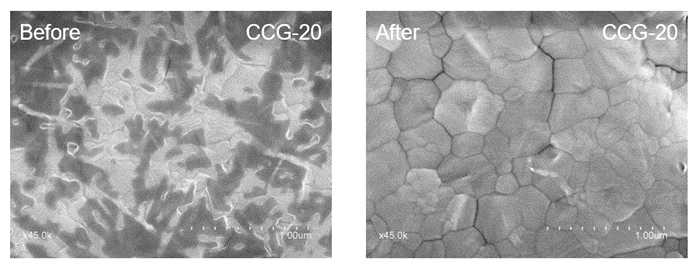


**Fig. S11** SEM images of tBTCA-modified perovskite films before and after washing with chlorobenzene. The dark regions that can be wash away can be assigned as tBTCA because of its soluble nature in chlorobenzene


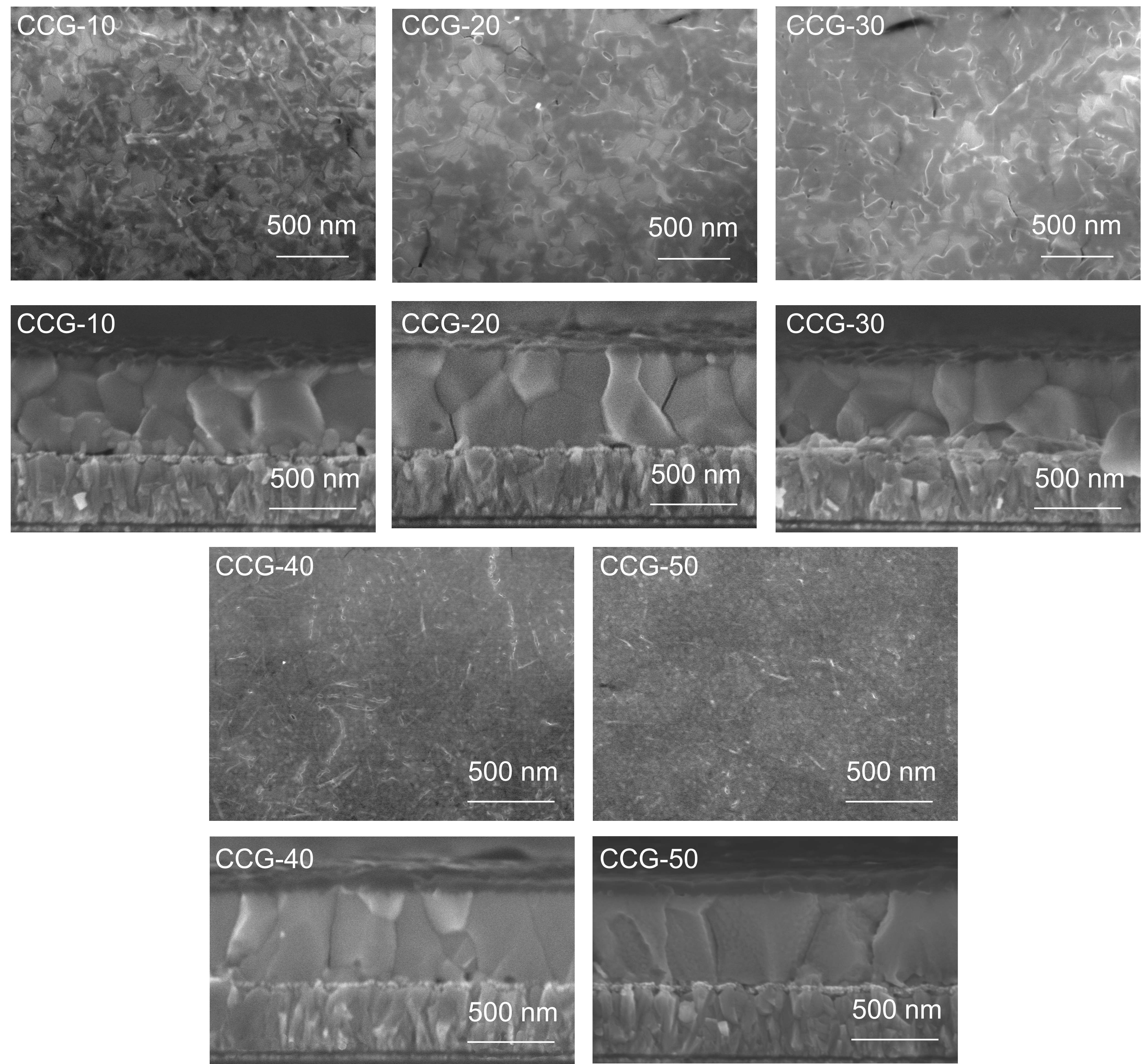


**Fig. S12** SEM and cross-sectional SEM images of tBTCA-modified perovskite films deposited on FTO/NiO_x_ substrates

**Fig. S13** Dependence of vertical perovskite grain size on the surface coverage ratio of the dark regions in SEM images


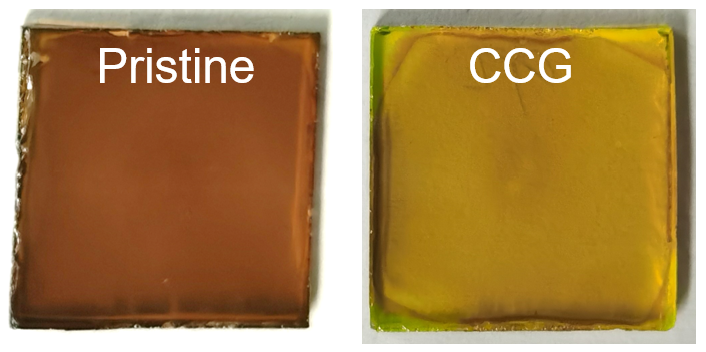


**Fig. S14** The photographs of the pristine and CCG precursor films after antisolvent dripping


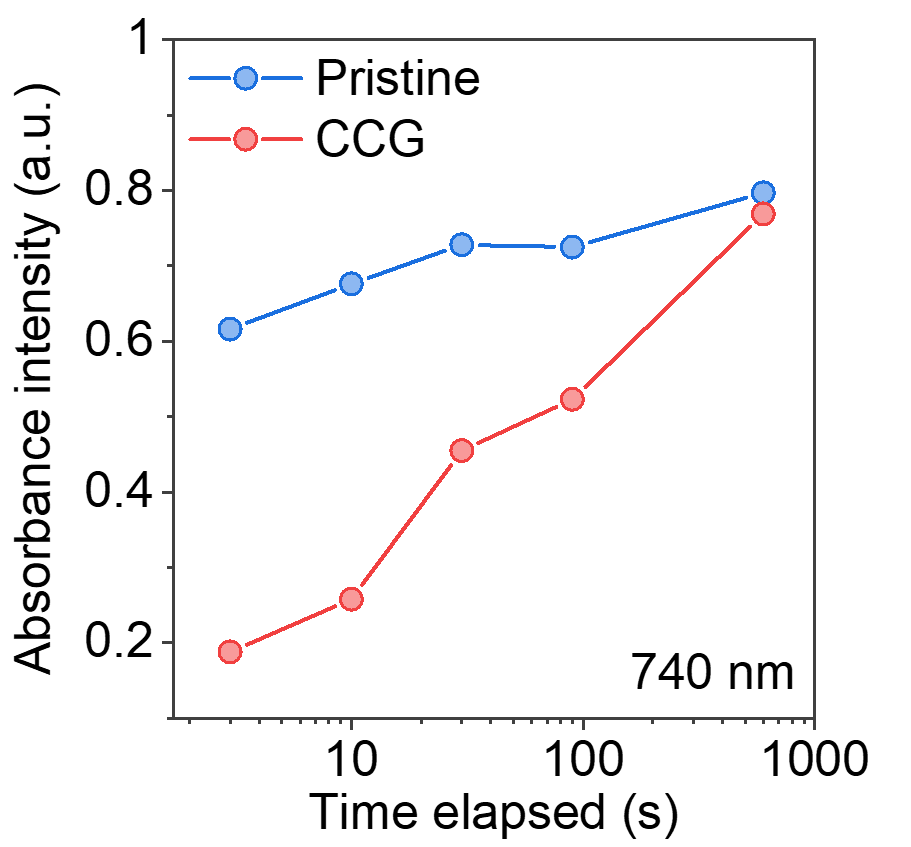


**Fig. S15** Evolution of absorbance at 740 nm of pristine and CCG films relative to annealing duration


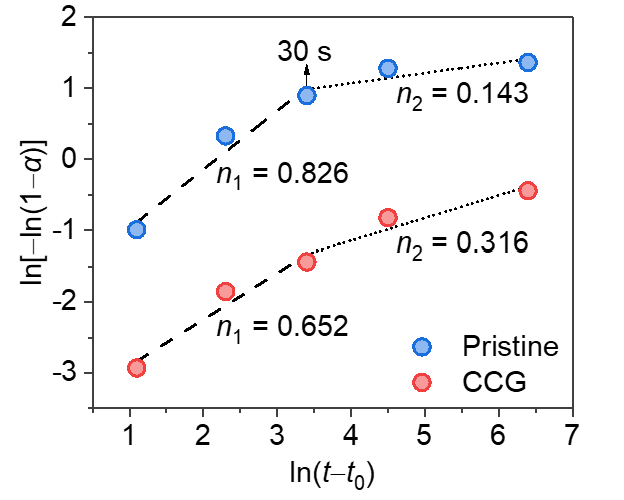


**Fig. S16** Diffraction intensity evolution of MAPbI_3_(110) plotted versus annealing duration by Sharp-Hancock presentation. The transition speed of intermediate-to-perovskite is positively correlated to the linearly fitted *n*-value


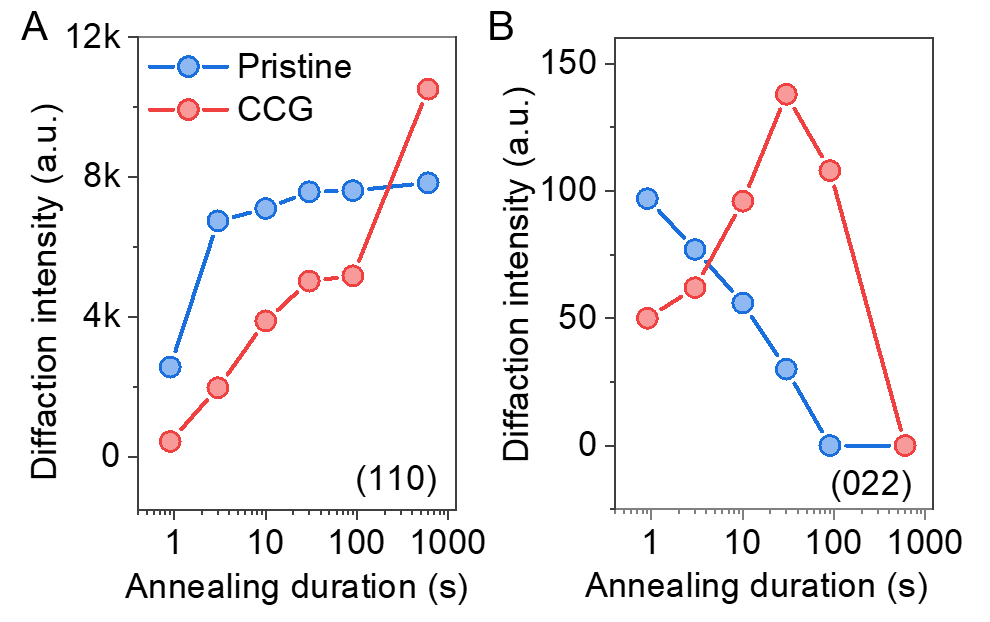


**Fig. S1****7** (**A**) MAPbI_3_(110) and (**B**) MA_2_Pb_3_I_8_(DMSO)_2_(022) plotted versus annealing duration


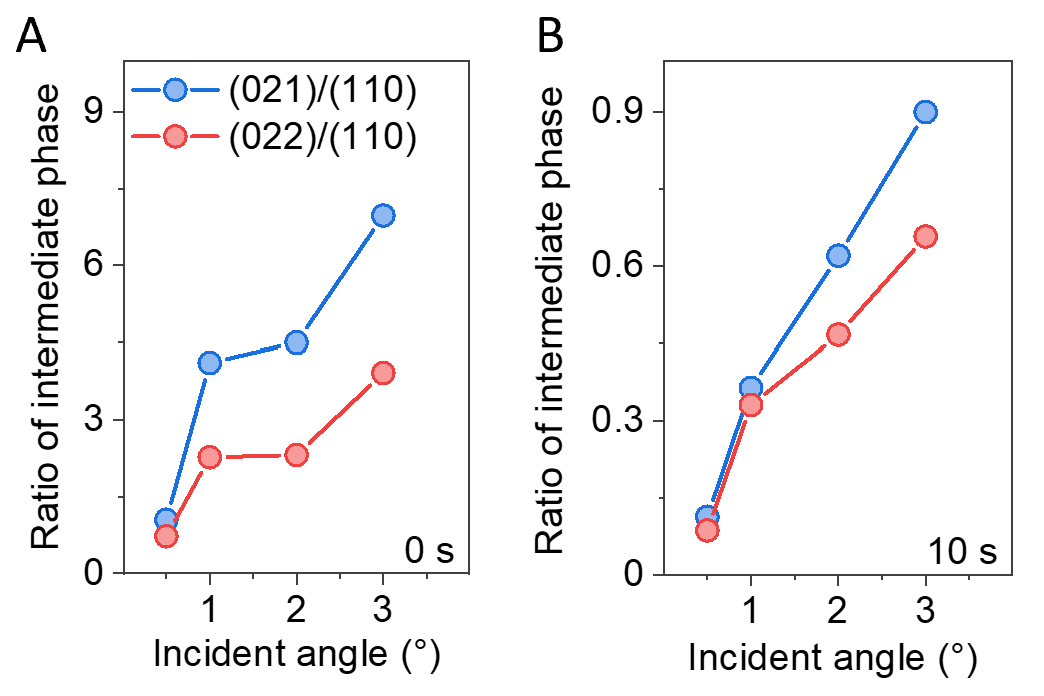


**Fig. S18** Percentage of intermediate phase to perovskite plotted versus the incident angle of GIXRD patterns for CCG films at annealing duration of (**A**) 0 s and (**B**) 10 s, respectively
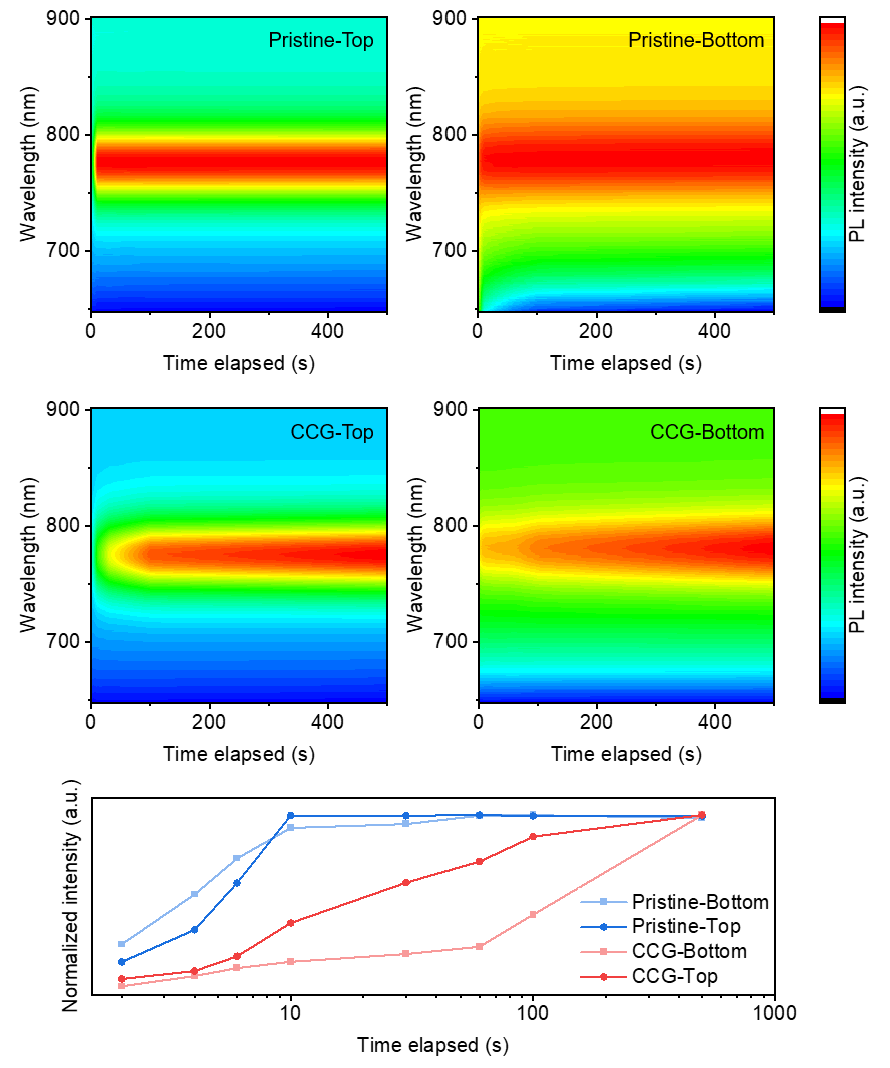


**Fig. S1****9** Time-dependent PL contour plot and intensity evolution of perovskite precursor films recorded under the excitation light from different directions. All films were annealed at 105 °C


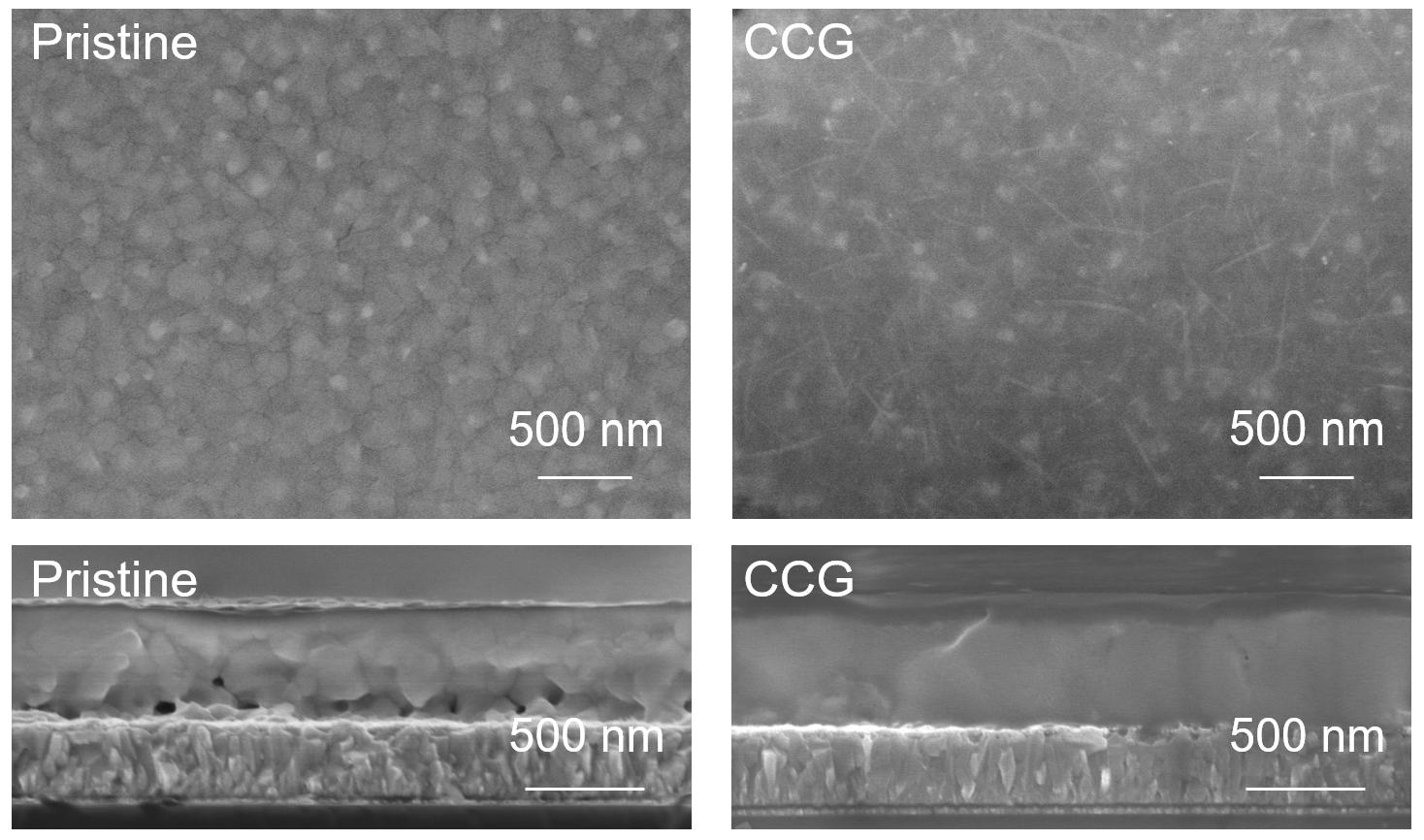


**Fig. S20** SEM and cross-sectional SEM images of pristine and CCG precursor films after antisolvent dripping without thermal annealing


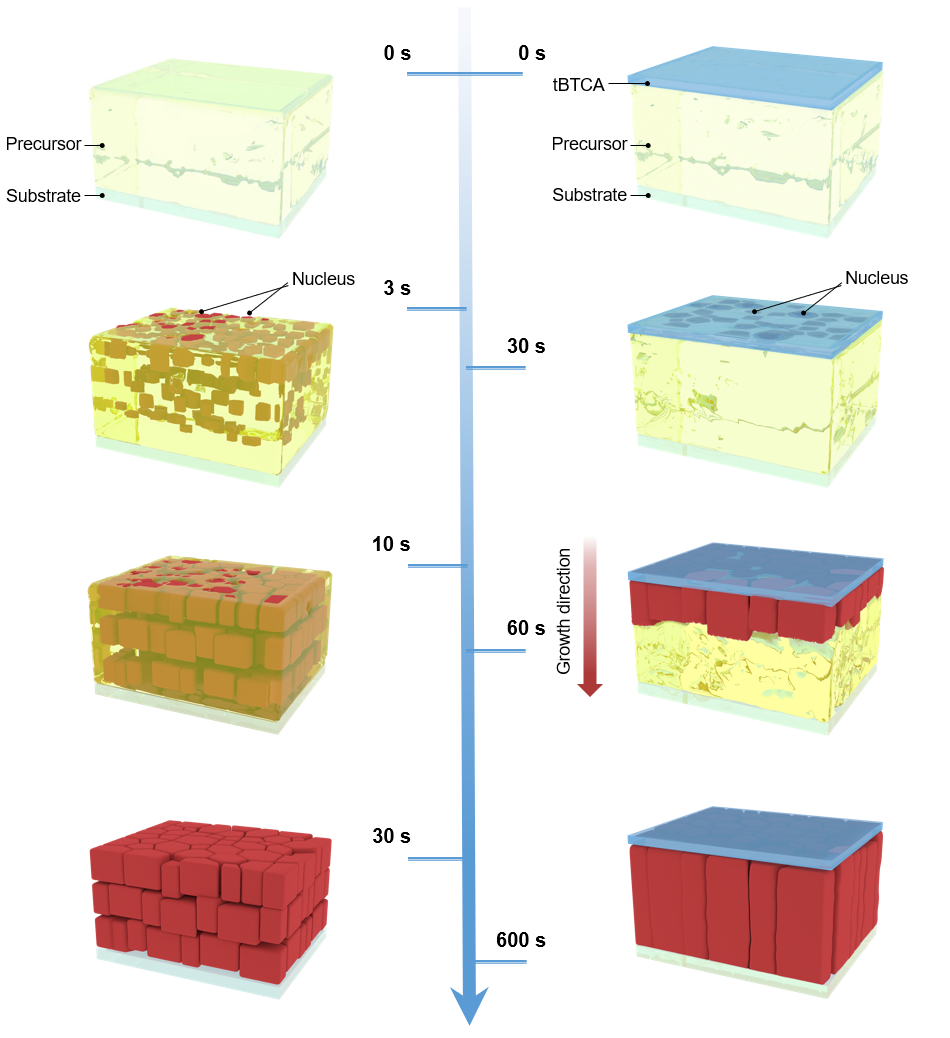


**Fig. S2****1** Schematic illustration of pristine and CCG perovskite formation process


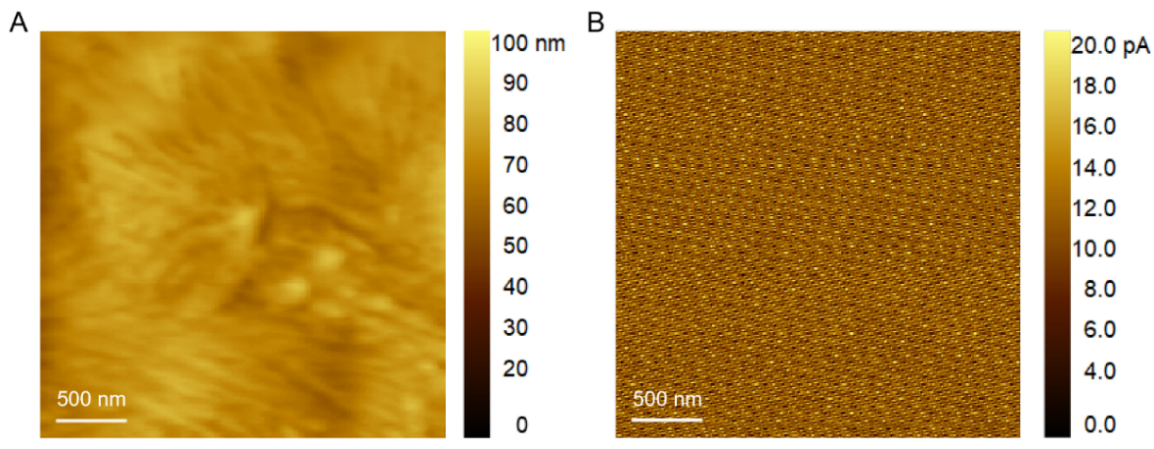


**Fig. S22** (**A**) AFM and (**B**) c-AFM images of CCG films with a tBTCA capping layer


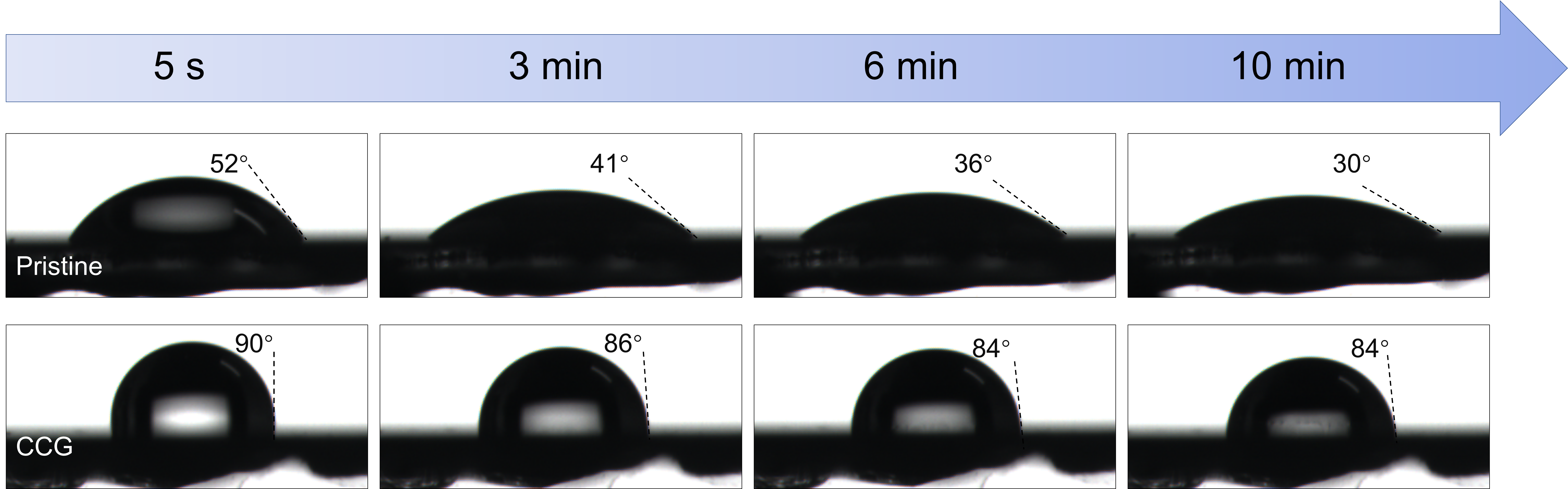


**Fig. S23** Water contact angle images of pristine and CCG films. The horizontal coordinate represents the loading time of water drops on different films


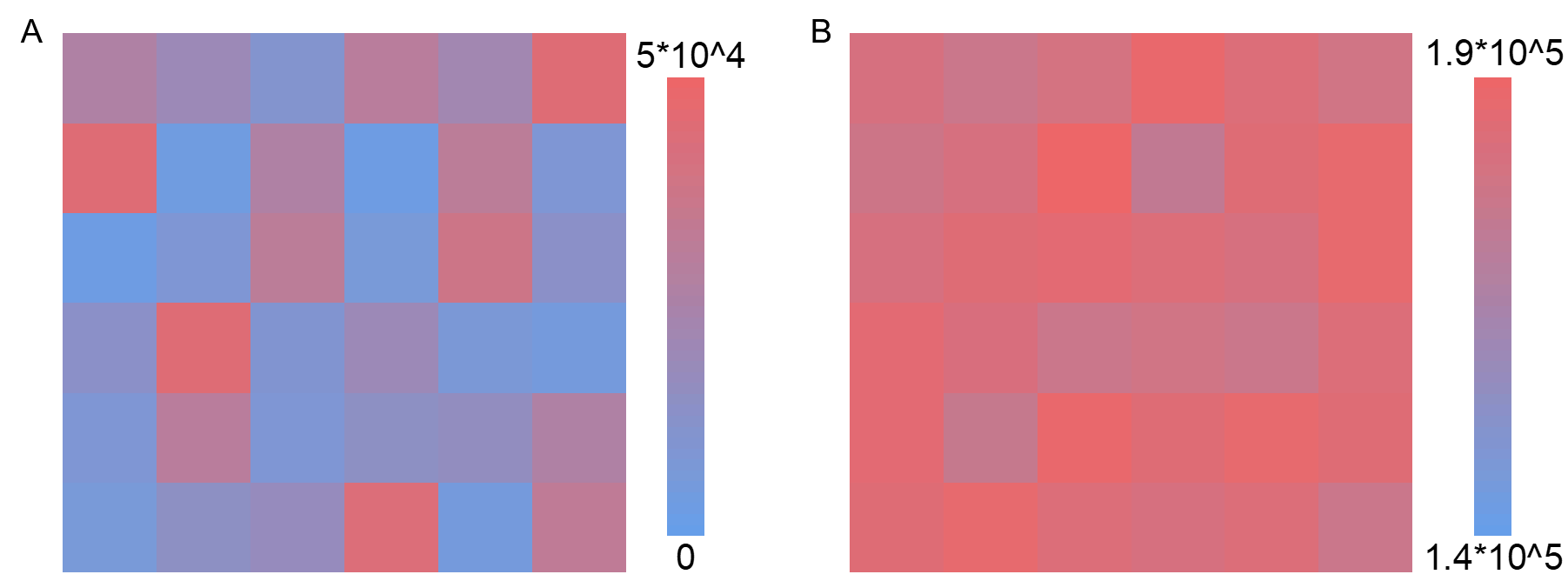


**Fig. 24** PL intensity thermogram of 36 perovskite films (**A**) without and (**B**) with CCG prepared in different batches


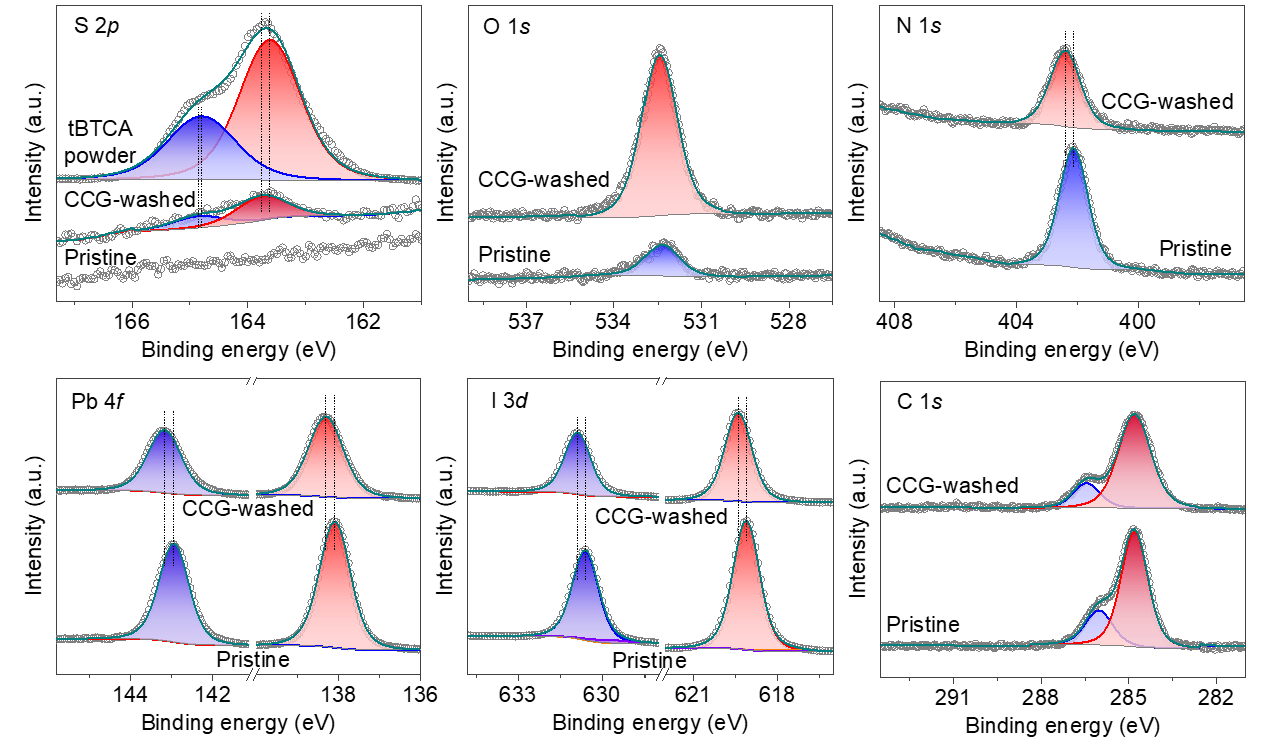


**Fig. S25** High-resolution XPS spectra of pristine and CCG films. The CCG layer was washed with chlorobenzene prior to measurement. The presence of XPS S 2*p*, O 1*s*, and C 1*s* peaks confirm the existence of bonded tBTCA at perovskite surface after washing treatment. The shift of Pb 4*f* peak a high-energy field for ~0.2 eV should be due to the presence of Pb−S and Pb−O bonds. The high-energy shift of ~0.3 eV of I 3*d* and N 1*s* XPS peaks should originate from the hydrogen bonding


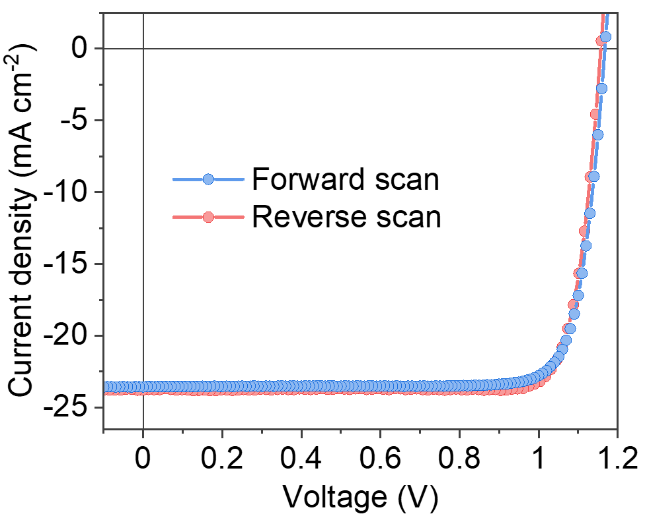


**Fig. S26** *J-V* curves of the champion CCG device measured in both reverse and forward scanning directions


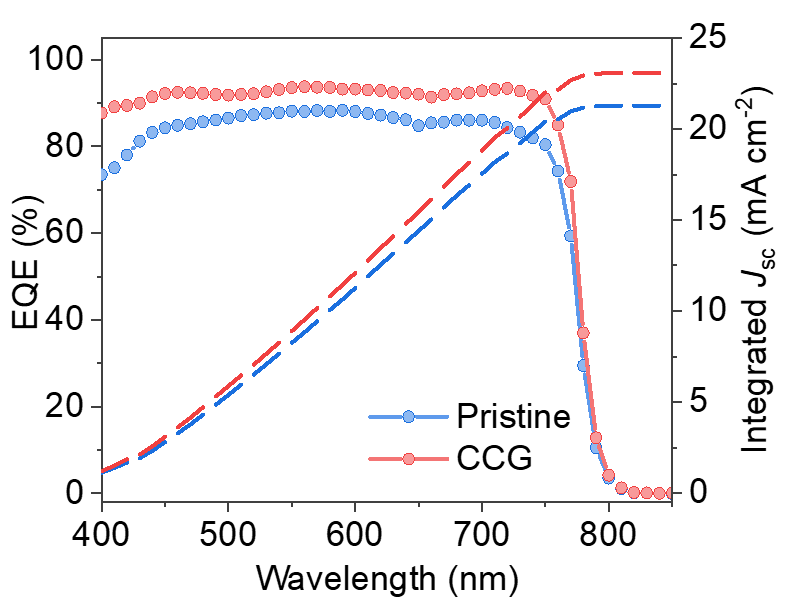


**Fig. S27** EQE spectra of pristine and CCG solar cells with the integrated *J*_SC_ determined to be 21.29 mA cm^−2^ and 23.08 mA cm^−2^, respectively


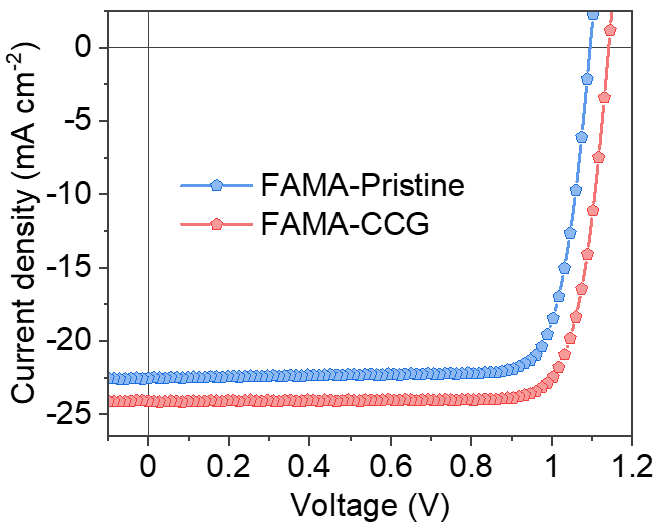


**Fig. S28** *J*-*V* curves of champion solar cells based on FAMA perovskite absorbers


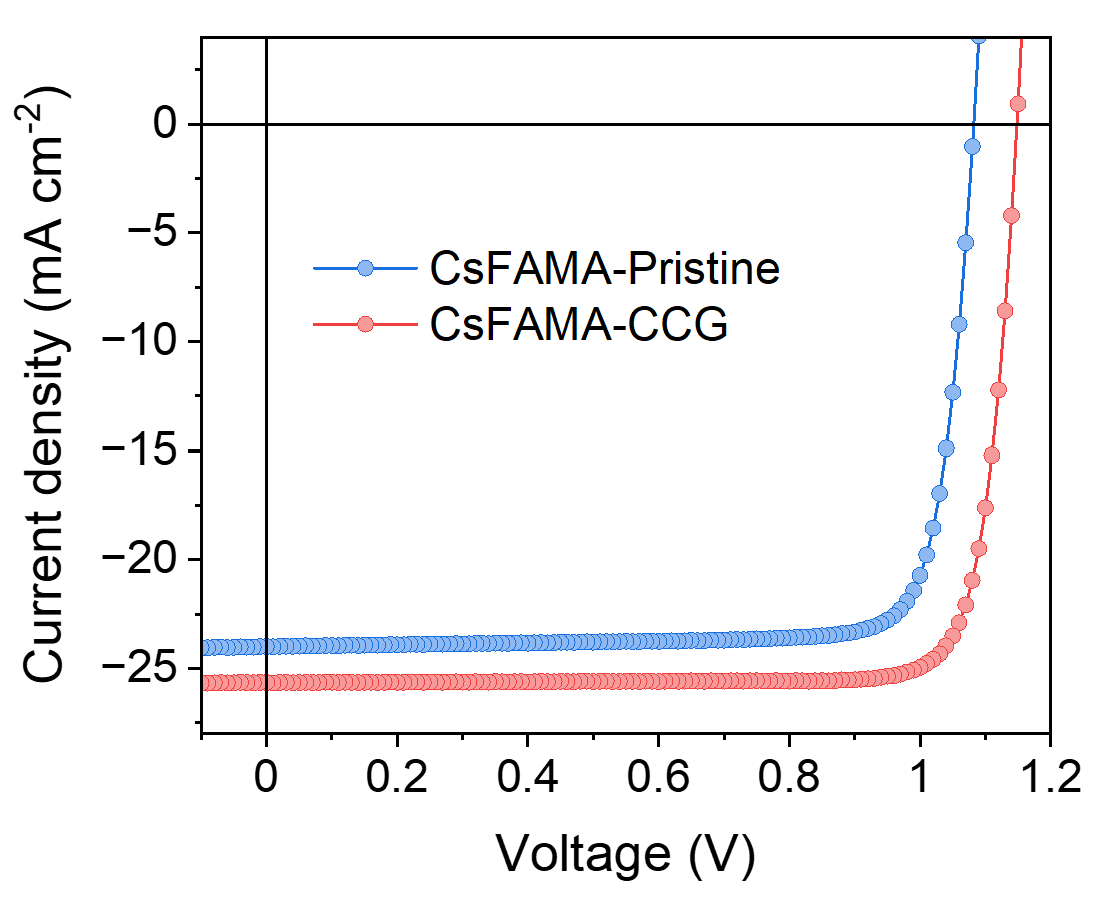


**Fig. S29** *J*-*V* curves of champion solar cells based on CsFAMA perovskite absorbers


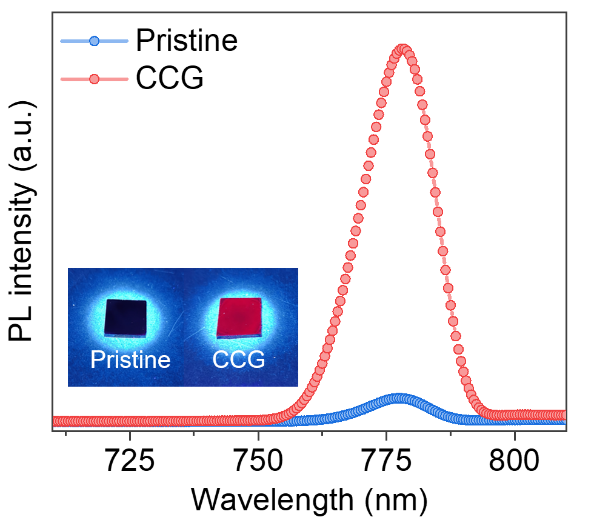


**Fig. S30** Steady-state PL spectra of pristine and CCG films deposited on quartz substrates. All PL spectra were acquired under the same exposure time and accumulation numbers. Inset: Photographs of pristine and CCG films (1.5 × 1.5 cm^2^) under UV light irradiation.


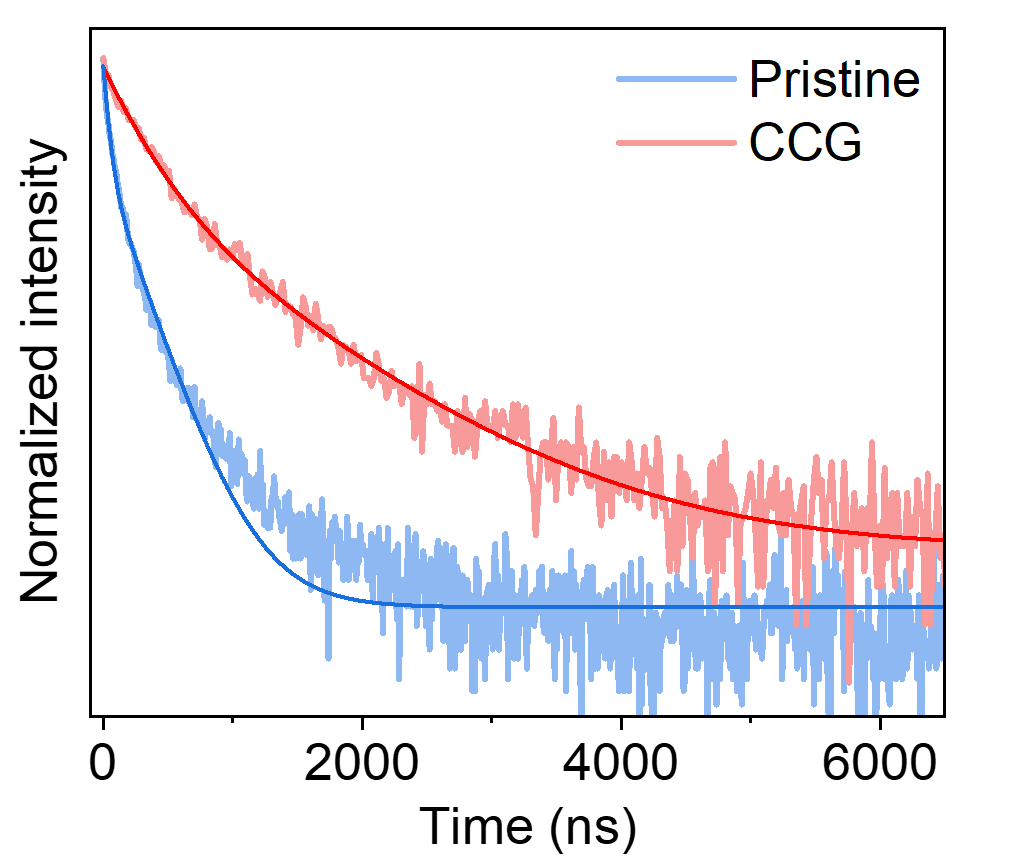


**Fig. S31** TRPL spectra of pristine and CCG films deposited on glass substrates


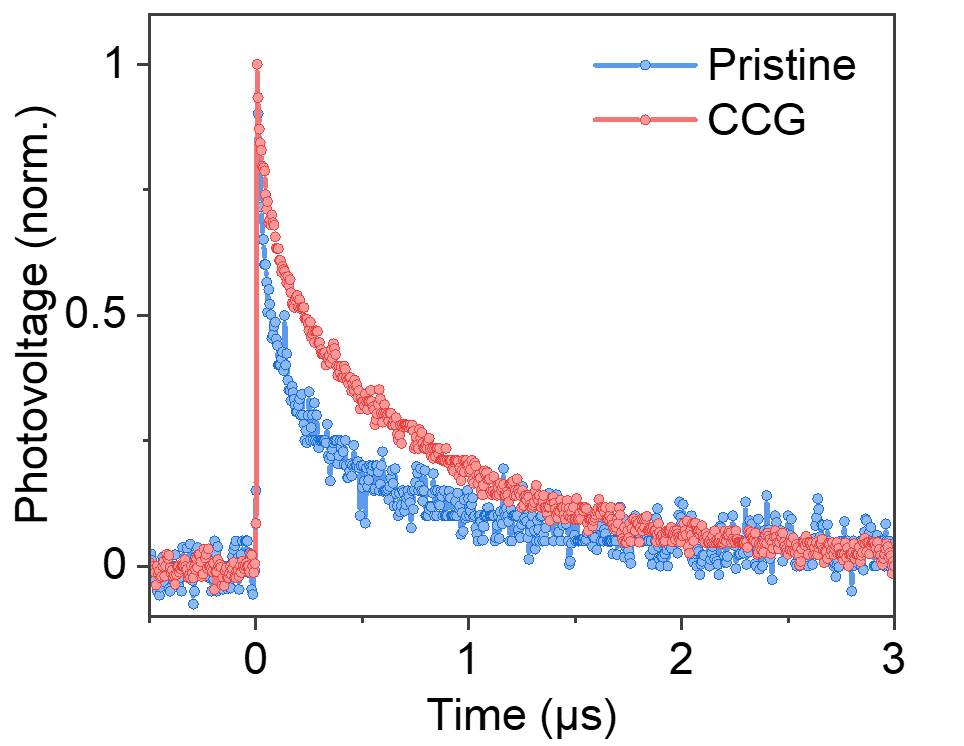


**Fig. S32** TPV decay curves of pristine and CCG solar cells under pulsed nanosecond laser


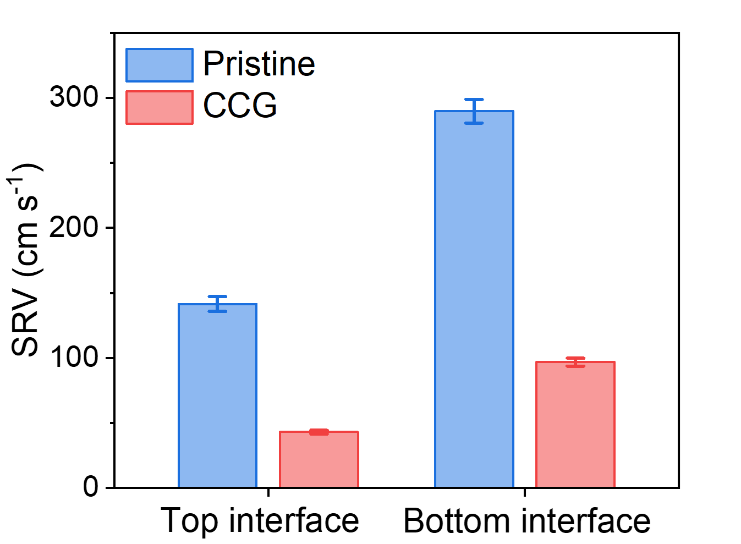


**Fig. S33** Calculated SRV of films deposited on glass and NiO_x_ substrates


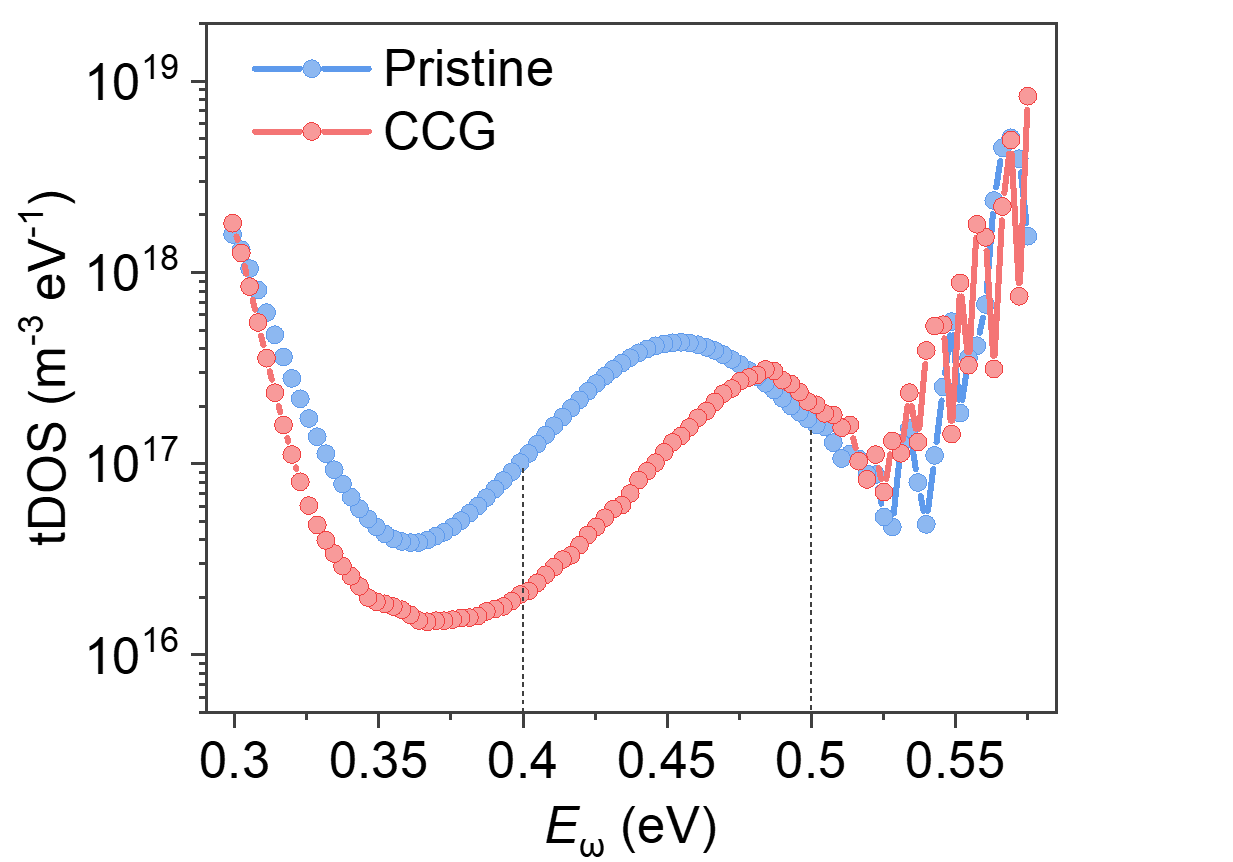


**Fig. S34** Trap density of pristine and CCG solar cells


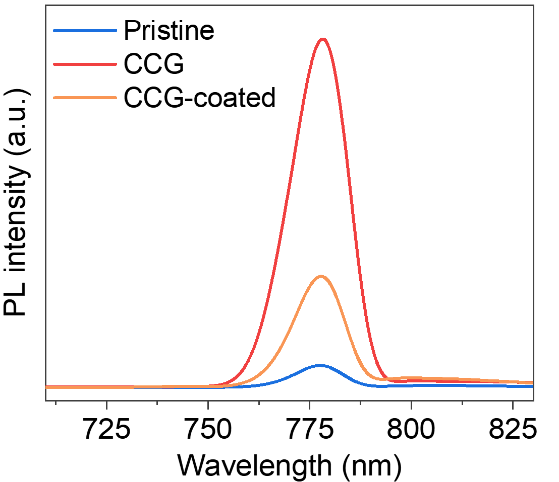


**Fig. S35** Steady-state PL spectra of pristine, CCG and CCG-coated films deposited on quartz substrates. The CCG-coated film was prepared by spin-coating tBTCA molecules on pristine perovskite surface


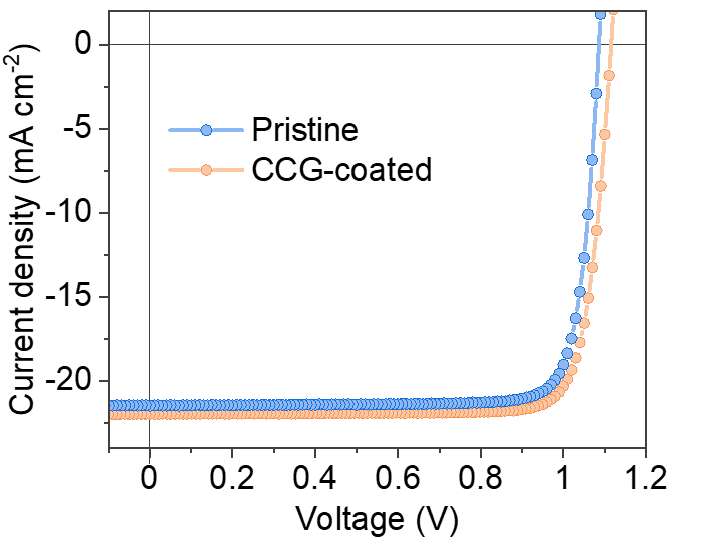


**Fig. S36** *J-V* curves of the champion solar cells based on pristine and CCG-coated films


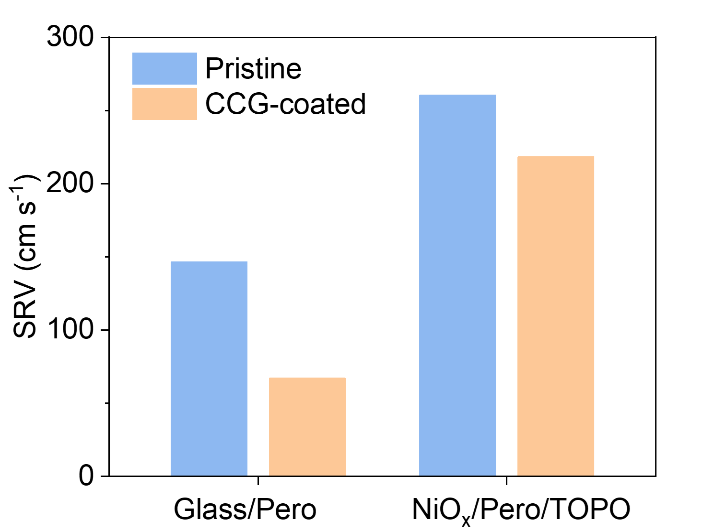


**Fig. S37** Calculated SRV of top and bottom interfaces for pristine and CCG-coated perovskite films


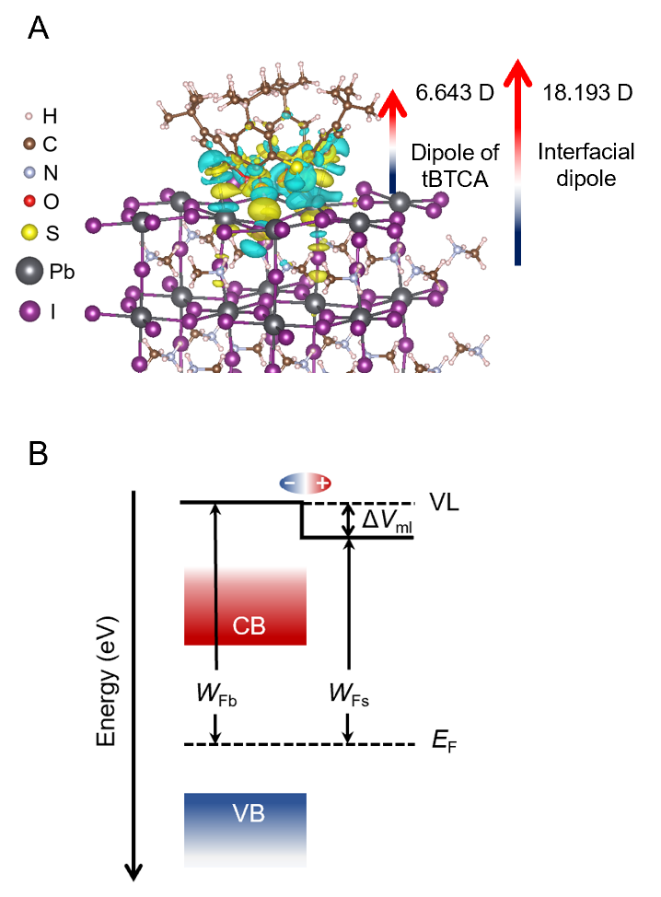


**Fig. S38** (**A**) Charge difference at the perovskite-supermolecule interface. Electrons accumulate to Pb−O, Pb−S, and H−I bonds, leaving the electron deficiency around O- and S-donors and hydrogen bond donors. The interfacial and molecular dipole moments are highlight by arrows. (**B**) Representation of the effect of the surface dipole on the perovskite band diagram for negative dipoles. VL is the local vacuum level, Δ*V*_ml_ is the vacuum level shift induced by the dipolar layers, *E*_F_ is the Fermi level energy, CB and VB are conduction and valence band, *W*_Fb_ is the bulk *W*_F_ and *W*_Fs_ is the surface *W*_F_


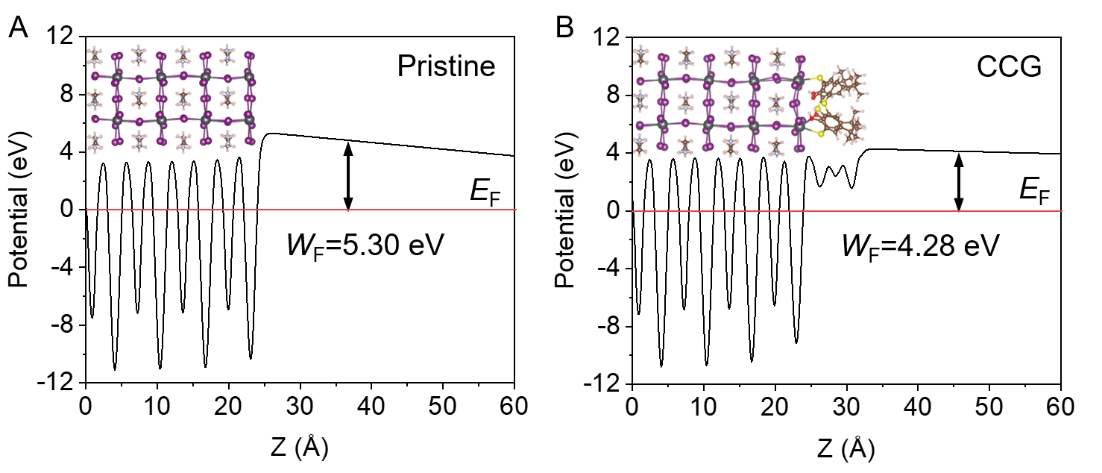


**Fig. S39** Plane-averaged ESP difference of perovskite slabs (**A**) without and (**B**) with tBTCA. The data is plotted geometrically match to the slab


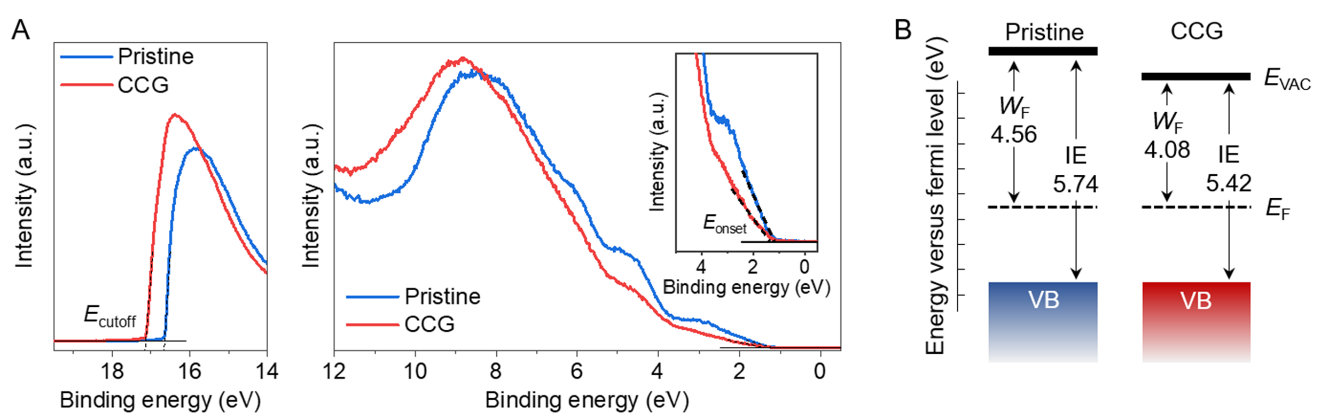


**Fig. S40** (**A**) UPS spectra and (**B**) as-determined energy level of pristine and CCG films. The CCG layer was washed with chlorobenzene prior to measurement


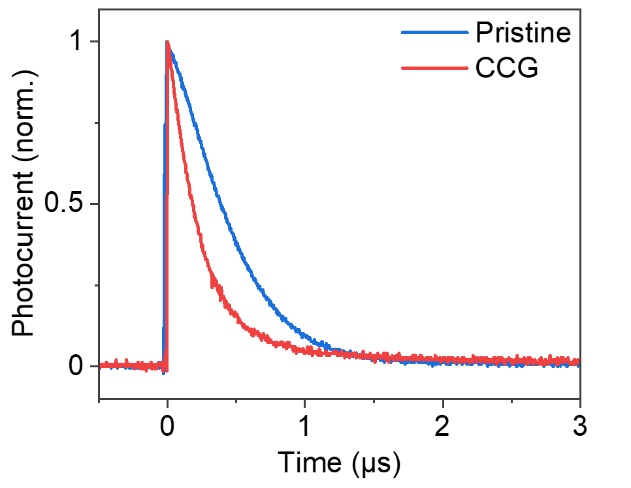


**Fig. S41** TPC decay curves of pristine and CCG solar cells under pulsed nanosecond laser


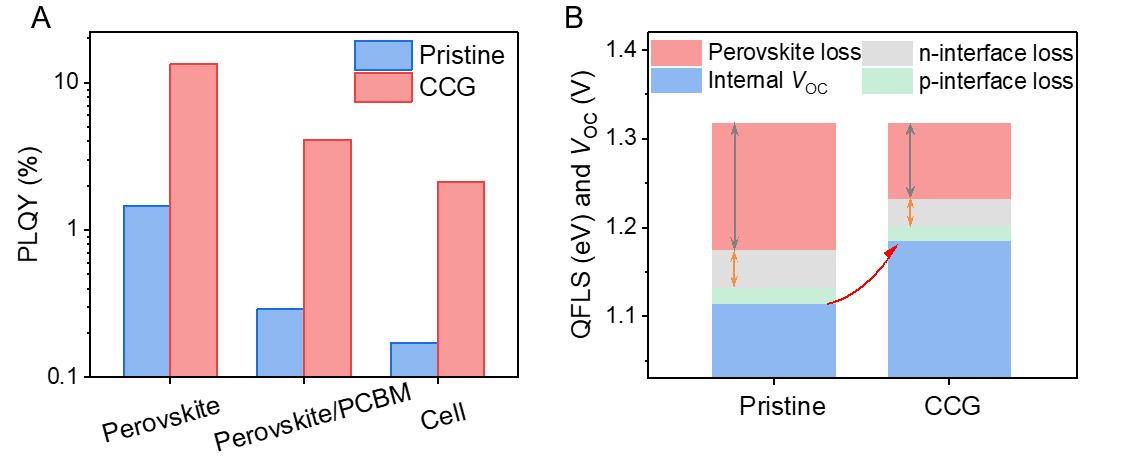


**Fig. S42** (**A**) PLQY diagram of perovskite film, perovskite/PCBM half stack, and full cell based on pristine and CCG perovskite active layers. (**B**) Voltage loss analysis for the pristine and CCG solar cells


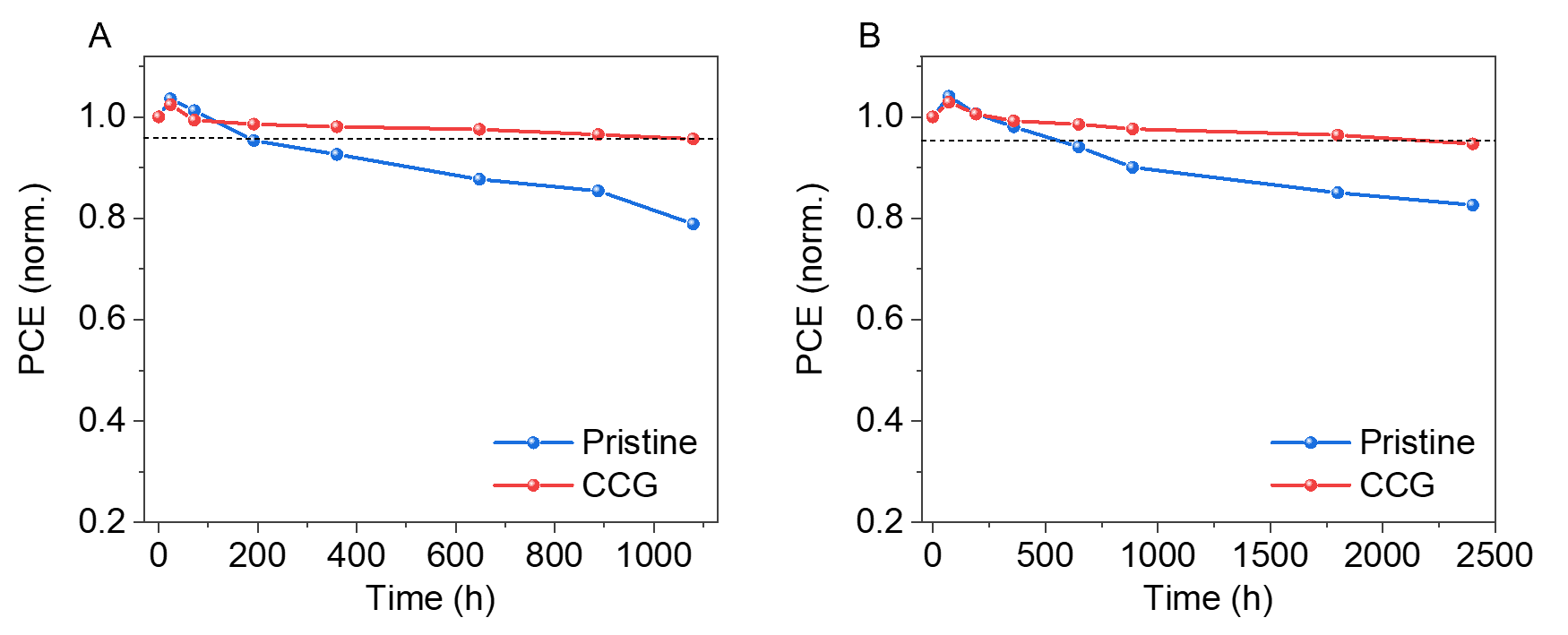


**Fig. S43** Long-term stability of the unencapsulated pristine and CCG devices under (A) continuous 100 mW cm**^−^**^2^ white LED illumination in nitrogen glove box and (B) ambient atmosphere with controlled humidity (RH, 25 ± 3%). All devices’ configuration is FTO/NiO_x_/perovskite/PC_61_BM/Au


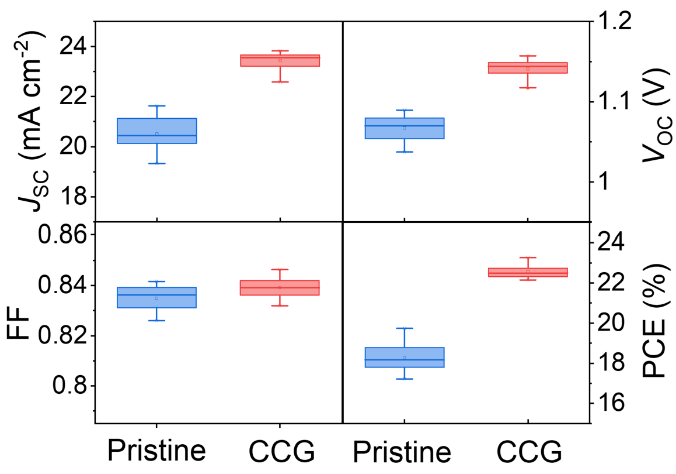


**Fig. S44** Comparison of photovoltaic metrics for 30 independent solar cells based on pristine and CCG perovskite films in the same batch


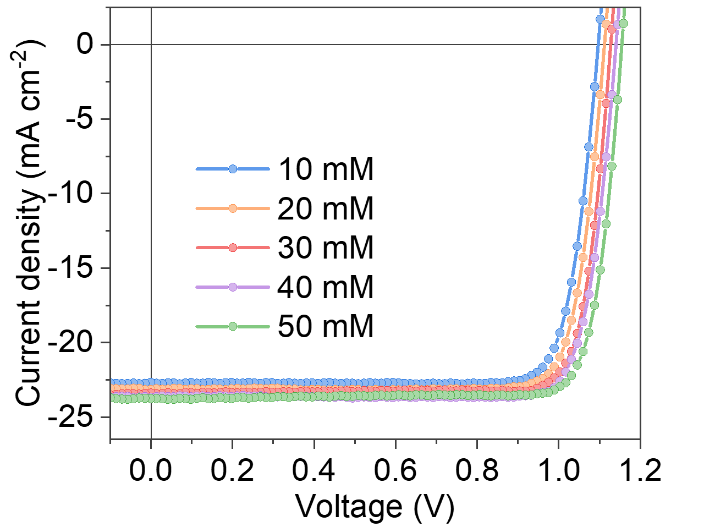


**Fig. S45** Influence of the tBTCA concentration on the *J*-*V* characteristics of perovskite solar cells


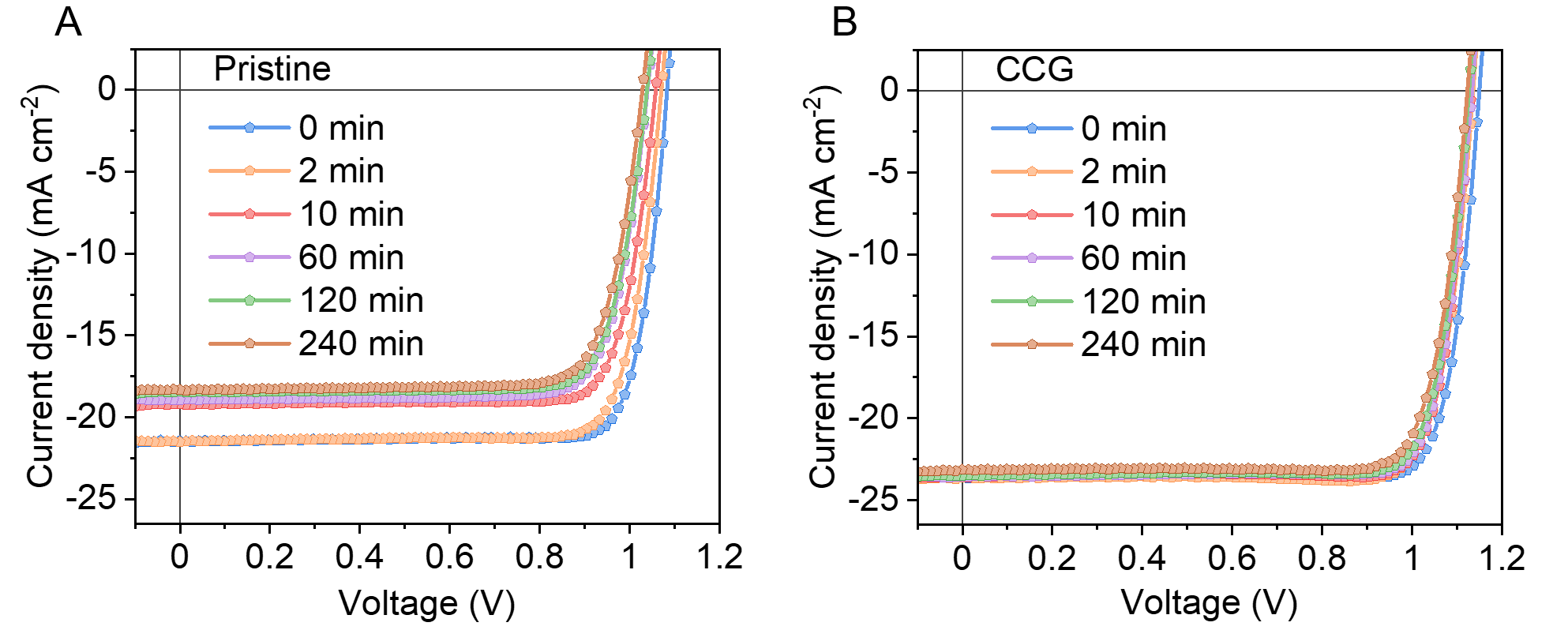


**Fig. S46** *J*-*V* curves of champion solar cells based on pristine and CCG films plotted versus annealing delay time

**Table S1** Summarized length and angle of H−I bonds between organic cations and inorganic matrix in geometrically optimized structure

| Label | Hydrogen bond | |
| --- | --- | --- |
|  | Bond length (Å) | Bond angle (°) |
| O(345)−H(193)−I(433) | 3.36 | 73 |
| O(346)−H(194)−I(437) | 3.09 | 93 |
| O(347)−H(195)−I(434) | 2.37 | 152 |
| O(348)−H(196)−I(444) | 3.26 | 87 |

**Table S2** Element contents of S and Pb in precursor films measured by ICP-OES. tBC[4]A is used for the CCG growth as it does not contain S element

|  | Pristine | CCG | MA_2_Pb_3_I_8_(DMSO)_2_ |
| --- | --- | --- | --- |
| S (mg/L) | 0.97 | 1.97 | / |
| Pb (mg/L) | 13.1 | 13.8 | / |
| S:Pb molar ratio | 0.48 | 0.92 | 0.67 |

**Table S3** Photovoltaic parameters of champion PSCs based on pristine and CCG films with varied tBTCA concentrations

| Absorber | tBTCA concentration  (mM) | *J*_SC_  (mA cm^−2^) | *V*_OC_  (V) | FF  (%) | PCE  (%) |
| --- | --- | --- | --- | --- | --- |
| Pristine | 0 | 21.58 | 1.09 | 84.04 | 19.77 |
| CCG | 60 | 23.78 | 1.16 | 84.53 | 23.32 |
| CCG-10 | 10 | 22.68 | 1.10 | 83.70 | 20.88 |
| CCG-20 | 20 | 23.15 | 1.11 | 83.70 | 21.51 |
| CCG-30 | 30 | 23.39 | 1.13 | 83.89 | 22.17 |
| CCG-40 | 40 | 23.68 | 1.14 | 84.36 | 22.77 |
| CCG-50 | 50 | 23.76 | 1.15 | 84.15 | 22.99 |

**Table S4** Photovoltaic parameters of champion solar cells based on FAMA-pristine and FAMA-CCG perovskite active layers

| Absorber | *J*_SC_  (mA cm^−2^) | *V*_OC_  (V) | FF  (%) | PCE  (%) |
| --- | --- | --- | --- | --- |
| FAMA-Pristine | 22.54 | 1.09 | 81.74 | 20.08 |
| FAMA-CCG | 24.11 | 1.14 | 82.19 | 22.59 |

**Table S5** Photovoltaic parameters of champion solar cells based on CsFAMA-pristine and CsFAMA-CCG perovskite active layers

| Absorber | *J*_SC_  (mA cm^−2^) | *V*_OC_  (V) | FF  (%) | PCE  (%) |
| --- | --- | --- | --- | --- |
| CsFAMA-Pristine | 24.00 | 1.08 | 83.50 | 21.69 |
| CsFAMA-CCG | 25.69 | 1.15 | 84.99 | 25.09 |

**Table S6** PLQY and QFLS results of perovskite film, perovskite/PCBM half stack, and full cell based on pristine and CCG perovskite active layers

| Sample | PLQY | QFLS (eV) | *V*_OC_ (V) |
| --- | --- | --- | --- |
| Perovskite | 0.0145 | 1.175 |  |
| Perovskite-CCG | 0.1341 | 1.233 |  |
| Perovskite/PCBM | 0.0029 | 1.133 |  |
| Perovskite-CCG/PCBM | 0.0411 | 1.202 |  |
| Cell | 0.0017 | 1.114 | 1.09 |
| Cell-CCG | 0.0213 | 1.185 | 1.16 |

**Table S7** Photovoltaic parameters of champion devices based on pristine and CCG films versus annealing delay time

| Absorber | Annealing delay time (min) | *J*_SC_  (mA cm^−2^) | *V*_OC_  (V) | FF  (%) | PCE  (%) |
| --- | --- | --- | --- | --- | --- |
| Pristine | 0 | 21.46 | 1.08 | 83.65 | 19.39 |
|  | 2 | 21.51 | 1.07 | 81.79 | 18.82 |
|  | 10 | 19.18 | 1.06 | 82.28 | 16.73 |
|  | 60 | 18.85 | 1.04 | 80.13 | 15.71 |
|  | 120 | 18.43 | 1.04 | 80.73 | 15.47 |
|  | 240 | 18.33 | 1.03 | 79.75 | 15.06 |
| CCG | 0 | 23.62 | 1.15 | 84.64 | 22.99 |
|  | 2 | 23.65 | 1.14 | 83.65 | 22.55 |
|  | 10 | 23.58 | 1.13 | 84.25 | 22.45 |
|  | 60 | 23.59 | 1.13 | 83.66 | 22.30 |
|  | 120 | 23.53 | 1.13 | 83.15 | 22.11 |
|  | 240 | 23.16 | 1.12 | 83.02 | 21.53 |

**Note S1** Formation mechanism analysis of perovskite film based on the classical nucleation theory.

The total Gibbs free energy (∆*G*_T_) of a system consists of two components, surface energy (∆*G*_S_) and free energy of bulk crystal (∆*G*_V_), which is given by [S1]:

$G_{T}=\Delta G_{S}+\Delta G_{V}$ (S1)

Assuming that the ∆*G*_T_ change of pristine and CCG films equals to each other under isothermal heating, nucleus will form mainly at the solid-air interface with increased ∆*G*_S_ and decreased ∆*G*_V_. The critical radius (*r*_C_) of nuclei that is relative to ∆*G*_V_ can be then determined by [S1, S2]:

$r_{C}=\frac{-2\gamma}{\Delta G_{V}}=\frac{2\gamma v}{k_{B}T\ln S}$ (S2)

where *r*_C_ corresponds to the minimum size at which a particle can survive in solution without being redissolved, *γ* is the surface tension that needs to break for nucleus formation, *v* is the molar volume, *k*_B_ is the Boltzmann constant, *T* is the temperature, *S* is the supersaturation of the solution. The tBTCA molecule should behave as a physical barrier layer to suppress the evaporation of solvent molecules, and therefore retain a high DMSO ratio and a relatively low *S* within the precursor film. Other parameters should be nearly identical under pristine and CCG conditions, therefore an increased *r*_C_ can be enabled for CCG film. As a result, the critical free energy (∆*G*_C_) to create the stable nuclei without undergoing redissolution is enhanced, which is given by [S2]:

$G_{C}=\frac{4}{3}\pi\gamma{r_{C}}^{2}$ (S3)

Accordingly, we conceptualize the crystallization process and the free energy change of pristine and CCG films. The molecule confinement enables gradual supersaturation of the system, followed by increasing nucleation energy barrier and the critical particle size. The stable nuclei further dominate their subsequent upward coarsening behavior, and ultimately lead to columnar perovskite grains [S3].

**Note S2** Surface recombination velocity (SRV) calculation.

The measured PL decay time constant τ is related to bulk lifetime τ_b_ and surface lifetime τ_s_ [S4].

$\frac{1}{\tau}=\frac{1}{\tau_{b}}+\frac{1}{\tau_{s}}$ (S4)

In the model, τ_b_ is a factor that depends on only the bulk material properties, while τ_s_ is related to the SRV, sample thickness (W), and diffusion constant (D) of the excess carriers. Given that any thin-film sample would have a top and bottom surface/interface, previous study has established that τ_s_ can be approximated analytically in two limiting cases.

First, when the SRVs at the top and bottom interfaces are identical, τ_s_ can be defined as follows:

$\tau_{s}\cong\frac{W}{2SRV}+\frac{1}{D}\left( \frac{W}{\pi} \right)^{2}$ (S5)

On the other hand, when the SRV at one contact (SRV_2_) is far greater than the SRV at the other contact (SRV_1_) or, equivalently, when SRV_1_ ≈ 0, τ_s_ can be defined as follows:

$\tau_{s}\cong\frac{W}{{SRV}_{2}}+\frac{4}{D}\left( \frac{W}{\pi} \right)^{2}$ (S6)

The bulk lifetime τ_b_ of perovskite films can be approximated by measuring TOPO-surface passivated films deposited on inert glass substrates [S5, S6]. The lifetimes of glass/perovskite samples increase by a factor of 9 from ~200 ns to ~1800 ns after TOPO treatment, suggesting that the lifetimes before TOPO passivation are limited by top surface recombination. In contrast, the lifetimes of films deposited on NiOₓ substrate are significantly shorter (~100 ns) and they do not increase after surface passivation by TOPO. The ~100 ns time scale decays are much longer than the typical charge transfer times (<5 ns). Therefore, these reduced lifetimes on NiOₓ are direct results of strong non-radiative interface recombination [S7]. Specifically, recombination is largely dominated by the NiOₓ/perovskite interface regardless of the top surface condition.

As discussed above, recombination in this work is either dominated by top surface (τ_top_ << τ_bottom_) when using glass substrates, or dominated by bottom NiOₓ interface (τ_bottom_ << τ_top_). We therefore adopted the asymmetric boundary condition.

Thickness W was measured by cross-sectional SEM images and diffusion coefficient D was obtained for each film by photoluminescence quenching method [S8]:

$D=\frac{L_{d}^{2}}{\tau}$ (S7)

where L_d_ is the diffusion length given by [S9]:

$L_{d}=\frac{2W}{\pi}\sqrt{2\left( \frac{\tau_{b}}{\tau_{quench}} \right)-1}$ (S8)

Finally, SRV is obtained by plugging W, D, τ_b_ and τ into the equation. We note that the SRV equation disentangles interface effect from bulk effect by treating bulk diffusion constant and bulk lifetime of each film as independent parameters. Therefore, SRV values accurately represent interface passivation effect alone. All values used for the calculation are summarized as follows:

| Lifetime (ns) | G/Pero | G/Pero  /TOPO | G/Pero  /Spiro | G/NiOx/Pero | | G/NiOx/Pero/TOPO | |
| --- | --- | --- | --- | --- | --- | --- | --- |
| Pristine | 296 | 1875 | 12 | 1.27 | 154 | 0.74 | 179 |
| CCG | 848 | 2328 | 10 | 0.92 | 443 | 0.86 | 472 |
|  | τ_b_ (ns) | τ_quench_ (ns) | W (nm) | D (m^2^/s) | | SRV (cm/s) | |
| Pristine | 1875 | 12 | 496±20 | 1.05±0.09 E-04 | | 141.50±5.71 | 289.89±9.12 |
| CCG | 2328 | 10 | 572±18 | 7.27±0.46 E-05 | | 42.94±1.35 | 96.78±3.05 |

**Note S3** Quasi-Fermi level splitting (QFLS) calculation.

QFLS is calculated by the following equation [S10, S11]:

$QFLS=k_{B}Tln(PLQY\frac{J_{G}}{J_{0, rad}})=k_{B}Tln(PLQY\frac{J_{G}}{q\int_{0}^{\infty} {EQE}_{PV}\left( E \right)\emptyset_{BB}\left( E \right)dE})$ (S9)

where *J*_G_ is the total free charge generation current density, *J*_0,rad_ is the radiative thermal equilibrium recombination current density in the dark and $\emptyset_{BB}$ is the black body radiative spectrum.

# Supplementary References

1. N.H. Fletcher, Size effect in heterogeneous nucleation. J. Chem. Phys. **29**(3), 572–576 (1958). <https://doi.org/10.1063/1.1744540>
2. N.T.K. Thanh, N. MacLean, S. Mahiddine, Mechanisms of nucleation and growth of nanoparticles in solution. Chem. Rev. **114**(15), 7610–7630 (2014). <https://doi.org/10.1021/cr400544s>
3. Y. Zhou, O.S. Game, S. Pang, N.P. Padture, Microstructures of organometal trihalide perovskites for solar cells: their evolution from solutions and characterization. J. Phys. Chem. Lett. **6**(23), 4827–4839 (2015). <https://doi.org/10.1021/acs.jpclett.5b01843>
4. A.B. Sproul, Dimensionless solution of the equation describing the effect of surface recombination on carrier decay in semiconductors. J. Appl. Phys. **76**(5), 2851–2854 (1994). <https://doi.org/10.1063/1.357521>
5. J. Wang, W. Fu, S. Jariwala, I. Sinha, A.K.Y. Jen et al., Reducing surface recombination velocities at the electrical contacts will improve perovskite photovoltaics. ACS Energy Lett. **4**(1), 222–227 (2019). <https://doi.org/10.1021/acsenergylett.8b02058>
6. I.L. Braly, D.W. DeQuilettes, L.M. Pazos-Outón, S. Burke, M.E. Ziffer et al., Hybrid perovskite films approaching the radiative limit with over 90% photoluminescence quantum efficiency. Nat. Photonics **12**(6), 355–361 (2018). <https://doi.org/10.1038/s41566-018-0154-z>
7. T. Kirchartz, J.A. Márquez, M. Stolterfoht, T. Unold, Photoluminescence-based characterization of halide perovskites for photovoltaics. Adv. Energy Mater. **10**(26), 1904134 (2020). <https://doi.org/10.1002/aenm.201904134>
8. S.D. Stranks, G.E. Eperon, G. Grancini, C. Menelaou, M.J.P. Alcocer et al., Electron-hole diffusion lengths exceeding 1 micrometer in an organometal trihalide perovskite absorber. Science **342**(6156), 341–344 (2013). <https://doi.org/10.1126/science.1243982>
9. E.M.Y. Lee, W.A. Tisdale, Determination of exciton diffusion length by transient photoluminescence quenching and its application to quantum dot films. J. Phys. Chem. C **119**(17), 9005–9015 (2015). <https://doi.org/10.1021/jp512634c>
10. M. Stolterfoht, P. Caprioglio, C.M. Wolff, J.A. Márquez, J. Nordmann et al., The impact of energy alignment and interfacial recombination on the internal and external open-circuit voltage of perovskite solar cells. Energy Environ. Sci. **12**(9), 2778–2788 (2019). <https://doi.org/10.1039/C9EE02020A>
11. J. Liu, S. Chen, D. Qian, B. Gautam, G. Yang et al., Fast charge separation in a non-fullerene organic solar cell with a small driving force. Nat. Energy **1**, 16089 (2016). <https://doi.org/10.1038/nenergy.2016.89>
